# Citizens Versus the Internet: Confronting Digital Challenges With Cognitive Tools

**DOI:** 10.1177/1529100620946707

**Published:** 2020-12-16

**Authors:** Anastasia Kozyreva, Stephan Lewandowsky, Ralph Hertwig

**Affiliations:** 1 Center for Adaptive Rationality, Max Planck Institute for Human Development; 2 School of Psychological Science, University of Bristol; 3 School of Psychological Science, University of Western Australia

**Keywords:** attention economy, behavioral policy, boosting, choice architecture, cognitive tools, decision aids, disinformation, false news, media literacy, nudging

## Abstract

The Internet has evolved into a ubiquitous and indispensable digital environment in which people communicate, seek information, and make decisions. Despite offering various benefits, online environments are also replete with smart, highly adaptive choice architectures designed primarily to maximize commercial interests, capture and sustain users’ attention, monetize user data, and predict and influence future behavior. This online landscape holds multiple negative consequences for society, such as a decline in human autonomy, rising incivility in online conversation, the facilitation of political extremism, and the spread of disinformation. Benevolent choice architects working with regulators may curb the worst excesses of manipulative choice architectures, yet the strategic advantages, resources, and data remain with commercial players. One way to address some of this imbalance is with interventions that empower Internet users to gain some control over their digital environments, in part by boosting their information literacy and their cognitive resistance to manipulation. Our goal is to present a conceptual map of interventions that are based on insights from psychological science. We begin by systematically outlining how online and offline environments differ despite being increasingly inextricable. We then identify four major types of challenges that users encounter in online environments: persuasive and manipulative choice architectures, AI-assisted information architectures, false and misleading information, and distracting environments. Next, we turn to how psychological science can inform interventions to counteract these challenges of the digital world. After distinguishing among three types of behavioral and cognitive interventions—nudges, technocognition, and boosts—we focus on boosts, of which we identify two main groups: (a) those aimed at enhancing people’s agency in their digital environments (e.g., self-nudging, deliberate ignorance) and (b) those aimed at boosting competencies of reasoning and resilience to manipulation (e.g., simple decision aids, inoculation). These cognitive tools are designed to foster the civility of online discourse and protect reason and human autonomy against manipulative choice architectures, attention-grabbing techniques, and the spread of false information.

In 1969, the year Neil Armstrong became the first person to walk on the moon, the precursor to the Internet—then known as ARPANET—was brought online. The first host-to-host message was sent from a computer at the University of California, Los Angeles, to a computer at Stanford University, and it read “lo”: The network crashed before the full message, “login,” could be transmitted. Fast forward half a century from this first step into cyberspace, and the Internet has evolved into a ubiquitous global digital environment, populated by more than 4.5 billion people and entrenched in many aspects of their professional, public, and private lives.

## The Role and Responsibility of Psychological Science in the Digital Age

The evolution of digital technologies has given rise to possibilities that were largely inconceivable in 1969, such as instant worldwide communication, mostly unfettered and constant access to information, democratized production and dissemination of information and digital content, and the ability to coordinate global political movements. The COVID-19 pandemic and resulting lockdowns serve as a striking example of just how indispensable the Internet has become to the global economy as well as to citizens’ well-being and livelihood. With much of the world stuck at home, the Internet is one of the most important tools for connecting with others, finding entertainment and information, and learning and working from home. But as the popular adage goes, there is no such thing as a free lunch. Digital technology has also introduced challenges that imperil the well-being of individuals and the functioning of democratic societies, such as the rapid spread of false information and online manipulation of public opinion (e.g., Bradshaw & Howard, 2019; Kelly et al., 2017), as well as new forms of social malpractice, such as cyberbullying (Kowalski et al., 2014) and online incivility (A. A. Anderson et al., 2014). Moreover, the Internet is no longer an unconstrained and independent cyberspace but, notwithstanding appearances, a highly controlled environment. Online, whether people are accessing information through search engines or social media, their access is regulated by algorithms and design choices made by corporations in pursuit of profits and with little transparency or public oversight. Government control over the Internet is largely limited to authoritarian regimes (e.g., China, Russia); in democratic countries, technology companies have accumulated unprecedented resources, market advantages, and control over people’s data and access to information (Zuboff, 2019).

**Fig. 1.**
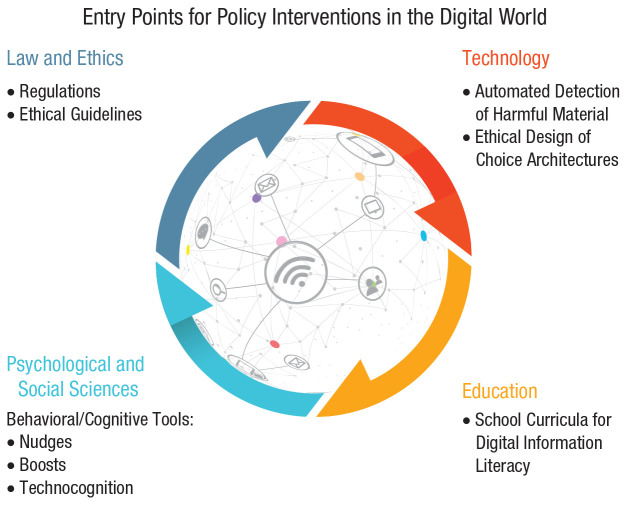
Entry points for policy interventions in the digital world: legal and ethical, technological, educational, and socio-psychological. Each entry point is shown with examples of potential policy measures and interventions. Entry points can inform each other; for instance, an understanding of psychological processes can contribute to the design of interventions for any entry point, and regulatory solutions can directly constrain and inform the design of technological and educational agendas. Icons are used under license from Adobe Stock.

This hidden commercial regulation has been brought into sharp focus by several scandals implicating the social-media giant Facebook in unethical dealings with people’s data (“The Cambridge analytica files,” 2018). Regulators and the general public have awakened to the extent to which digital technologies and tech companies can infringe on people’s privacy and control access to information. Furthermore, these scandals have revealed the manipulative power of techniques such as “dark ads” (advertising messages that are visible only to those who are targeted by them) and microtargeting (customizing advertisements to particular individuals), which are meant to influence people’s decision-making and voting behavior, by exploiting their psychological vulnerabilities and personal identities (e.g., Matz et al., 2017). There is clearly no panacea for solving these problems. Instead, there are multiple entry points for addressing the existing and emerging challenges (Fig. 1; see also Lazer et al., 2018). We argue that psychological science is indispensable in the analysis of the key challenges to human cognition and decision-making in the online world but also in the design of ways to respond to them.

The first entry point for interventions comes from the normative realm of law and ethics; this includes legislative regulations and ethical guidelines—for example, ethics guidelines for trustworthy AI by the High-Level Expert Group on Artificial Intelligence (2019) or the European Union (EU) Code of Practice on Disinformation (European Commission, 2018); for an overview of misinformation legislative actions worldwide, see also Funke and Flamini (2019). Regulatory interventions can, for instance, introduce transparent rules for data protection (e.g., the EU’s General Data Protection Regulation [GDPR]; European Parliament, 2016) or for political campaigning on social media and can impose significant costs for violating them; they can also implement serious incentives (and disincentives) for tech firms and the media to ensure that shared information is reliable and online conversation is civil. Regulatory initiatives should strive to create a coherent user-protection framework instead of the fragmentary legislative landscape currently in place (e.g., for Germany and the EU, see Jaursch, 2019).^1^

The second entry point for interventions is technological: Structural solutions are introduced into online architectures to mitigate adverse social consequences. For example, social-media platforms can take technological measures to remove fake and automated accounts, ensure transparency in political advertisement, and detect and limit the spread of false news using automated or outsourced fact-checking (e.g., Harbath & Chakrabarti, 2019; G. Rosen et al. 2019). However, such measures are mainly self-regulatory, depend heavily on the company’s good will, and are often introduced only after considerable public, political, and regulatory pressure.

The third entry point for interventions is educational. These interventions are directed at the public as recipients and producers of information—for example, school curricula for digital-information literacy that teach students how to search, filter, evaluate, and manage data, information, and digital content (e.g., Breakstone et al., 2018; McGrew et al., 2019).

Finally, the fourth entry point for interventions comes from psychological and social sciences and includes behavioral and cognitive interventions: Here, nonregulatory, nonmonetary policy measures are implemented to empower people and steer their decision-making toward greater individual and public good. In online behavioral and cognitive policy making, which is the main focus of this article, there are three notable approaches to designing interventions. The first is *nudging*, which aims to guide people’s behavior through the design of choice architectures (Thaler & Sunstein, 2008). The second is *boosting*, which seeks to improve people’s cognitive and motivational competencies (Hertwig & Grüne-Yanoff, 2017). The third is *technocognition*, which aims to design technological solutions resting on and informed by psychological principles identified in the study of human cognition (Lewandowsky, Ecker, & Cook, 2017).

These four entry points for interventions—coming from law, technology, education, and psychological science—are interrelated, and they can and should inform each other. For example, regulations on the ethical design of digital technologies should inform technological, educational, and behavioral interventions. Moreover, understanding psychological processes is essential for all four approaches; for instance, behavioral and cognitive insights can be useful for designing both educational and technological tools as well as regulatory interventions.

In this article we are concerned specifically with behavioral and cognitive interventions. Our main aim is to identify key challenges to people’s cognition and behavior in online environments and then to present a conceptual map of our preferred cognitive intervention: boosting. We focus on boosts for several reasons. First, we hold that the philosophy of the Internet is one of empowerment (e.g., Deibert, 2019; Diamond, 2010; Khazaeli & Stockemer, 2013). This is reflected in the EU’s approach, which highlights citizen empowerment as a goal of European digital policy (European Commission, 2020). The president of the European Commission echoed this sentiment, stating that “Europe’s digital transition must protect and empower citizens, businesses and society as a whole” (von der Leyen, 2020, para. 11). Second, although the call to increase people’s ability to deal with the challenges of online environments is growing louder (e.g., Directorate-General for Communications Networks, Content and Technology, 2018; Lazer et al., 2018), there has been no systematic account of interventions based on insights from psychological science that could form the foundation of future efforts. Third, the Internet is a barely constrained playground for commercial policy makers and choice architects acting in accordance with financial interests; in terms of power and resources, benevolent choice architects in the public sector are at a significant disadvantage. It is therefore crucial to ensure that psychological and behavioral sciences are employed not to manipulate users for financial gain but instead to empower the public to detect and resist manipulation. Finally, and crucially, boosts are probably the least paternalistic measures in the toolbox of public-policy makers and potentially the most resilient in the face of rapid technological change, in that they aim to foster lasting and generalizable competencies in users.

We begin by comparing online and offline environments to prepare the ground for considering the impact that new digital environments have on human cognition and behavior (Systematic Differences Between Online and Offline Environments section). Second, we consider the challenges that people encounter in the digital world and show how they affect users’ cognitive and motivational abilities. We distinguish four types of challenges: persuasive and manipulative choice architectures, AI-assisted information architectures, false and misleading information, and distracting environments (Challenges in Online Environments section). Third, we turn to the question of how to counteract these challenges. We briefly review the types of behavioral and cognitive interventions that can be applied to the digital world (Behavioral Inventions Online: Nudging, Technocognition, and Boosting section). We then identify four types of cognitive tools: *self-nudging*, which aims at enhancing people’s agency in their digital environments; *deliberate ignorance*, which can be used as a tool for information management; *simple decision aids*, which can help people accurately assess content they encounter online; and *inoculation*, a preemptive intervention that aims to boost people’s resilience to online misinformation and manipulation (Boosting Cognitive Competencies in Online Environments section). These tools are designed to foster the civility of online discourse and protect reason and human autonomy against manipulative choice architectures, attention-grabbing techniques, and the spread of false information. We conclude with a brief discussion of how psychological science can help create an Internet for citizens.

## Systematic Differences Between Online and Offline Environments

The Internet and the devices people use to access it represent not just new technological achievements but also entirely new artificial environments. Much like people’s physical surroundings, these are environments in which people spend time, communicate with each other, search for information, and make decisions. Yet the digital world is a recent phenomenon: The Internet is 50 years old, the Web is 30, and the advanced social Web is merely 15 (for definitions, see Table 1). New adjustments and features are added to these environments on a continuous basis, making it nearly impossible for most users, let alone regulators, to keep abreast of the inner workings of their digital surroundings.

**Table 1.** Glossary of Technical Terms

Term	Definition
AI-assisted information architectures	AI-powered algorithmic tools that filter and mediate information online (e.g., targeted advertising, personalized recommender systems, algorithmic filtering in search engines, personalized curation of news feeds on social media; see Figure 3 for an overview).
Algorithm	In the context of digital environments, a computer program that orders, classifies, generalizes, predicts, and filters information online. Algorithms can be rule-based (i.e., instructions are programmed by humans) or self-learning programs (“machine learning”).
Artificial intelligence (AI)	In the context of digital environments, mainly refers to self-learning computer programs (“machine learning”) that analyze people’s personal data and digital footprints to customize their online experience. Also includes fields such as robotics, knowledge representation and reasoning, planning, and computer vision.
Boost	Cognitive intervention that aims to foster people’s competencies. Boosts target cognition (e.g., simple rules for online reasoning) and the environment (e.g., pop-ups with information about the online source).
Choice architect	Designer of choice environments; can be public or commercial. A public choice architect is a policy maker who uses insights from behavioral science and other sources to alter people’s choice environments to achieve behavioral change. Benevolent public choice architects do not act with the goal of maximizing revenue (unlike most commercial choice architects) but rather with the goal of maximizing individual and collective well-being.
Choice architecture	Design of the external environments within which people make decisions (e.g., location of subway exits, presentation of foods in a cafeteria, display of search results for local restaurants on Google Maps, privacy settings on Facebook). Strategically organizing the external context in which people make decisions is one way to affect their choices.
Dark pattern	Design of user interfaces employed to steer people’s choices toward unintended decisions in the service of commercial interests.
Data privacy	Online, a set of rules for how Internet companies collect, share, and use personal information. An important aspect of data privacy concerns users’ choice to reveal or protect their personal information.
Internet	A global system of interconnected computer networks that includes several applications—e.g., the Web, e-mail, messenger systems, and mobile applications—for communication between devices and for access to the information contained within these networks.
Nudge	Behavioral intervention in the choice architecture that alters people’s behavior in a predictable way (e.g., default privacy settings). Educative nudges remind and prompt behavior (e.g., by providing additional useful information like fact-check labels).
Personalized or targeted advertising	A type of online advertising that shows ads to people on the basis of their online activity as well as both stated and inferred characteristics (e.g., gender, age, interests, political views, personality traits).
Recommender (also: Recommendation) system	Information-filtering and associations-finding algorithm that suggests products based on users’ past activities and preferences as well as the activities and preferences of other users with similar tastes.
Social web	Web-based social networks and other collaborative platforms (e.g., forums). Advanced social web: global social-media websites (e.g., Facebook, Twitter).
Technocognition	Cognitively inspired technological intervention in information architectures (e.g., introducing friction in the process of sharing offensive material).
Web (a.k.a. World Wide Web or www)	A standardized system for accessing and navigating information on the Internet; requires web browsers for access.

Online reality tends to be seen as different from the physical world, and computer-mediated social activities are often described as inferior substitutes for real-life or face-to-face interactions (for an overview, see Green & Clark, 2015). However, this presumed dualism between online and offline worlds is becoming more problematic—and possibly obsolete—as the line separating the two environments continues to blur. The ubiquitous nature of computing^2^ and the integration of digital devices and services into material objects (e.g., cars) and actions in the physical world (e.g., navigation) make it difficult to delineate when one is truly online or offline—a phenomenon that Floridi (2014) called the “onlife experience” (p. 43). This effect is highly visible in computerized work environments, where more and more of people’s working time is spent online. According to a report by the European Commission (2017), the use of digital technologies has increased significantly in the past 5 years in more than 90% of workplaces in the EU, and most jobs now require at least basic computer skills.

That said, the digital world differs from its offline counterpart in ways that have important consequences for people’s online experiences and behavior. We will proceed by outlining several ways in which online ecologies do not resemble offline environments. A systematic understanding is required not only to fill the gaps in knowledge of the psychologically relevant aspects of the digital world but also to ensure that psychological interventions take into account the specifics of these new environments and the particular challenges that people are likely to face there. First steps have already been made. Marsh and Rajaram (2019) identified 10 properties of the Internet—including accessibility, unlimited scope, rapidly changing content, and inaccurate information—that they organized into three categories: (a) content (what information is available), (b) Internet usage (how information is accessed), and (c) the people and communities that create and spread the content (who drives information). They argued that these properties can affect cognitive functions, such as short-term and long-term memory, and reading, and have an effect on social influence online. Other relevant classifications summarizing the differences between online and offline environments in the context of social media include those provided by McFarland and Ployhart (2015)^3^ and Meshi et al. (2015).^4^

We expand on these classifications by focusing on two broad types of differences between online and offline ecologies: differences in structure and functionality and differences in perception and behavior (i.e., how people perceive the online and offline worlds and how their behavior might differ accordingly). A list of characteristics of online environments can be found in Table 2, which is followed by a detailed discussion of each characteristic.

**Table 2.** Characteristics of Online Environments

Structure and functionality	Perception and behavior
Group sizes	Social cues and communication
Amount of information, limitless space and storage	Reliability of information and cues for epistemic quality
Rapid change and adaptivity	Social calibration
Intelligence, personalization, and datafication	Self-disclosure and privacy behavior
Choice architectures and the power of design	Norms of civility
	Perception of reality

### Differences in structure and functionality

#### Group sizes

In 2020, there are more than 4.6 billion people (or almost 60% of the global population) and around 30 billion devices connected to the Internet.^5^ Digital technologies have changed the public sphere, connecting people separated in both time and space and creating the “digital public” (Bunz, 2014). Indeed, one of the predominant uses of the Internet is for communication. The social Web boasts impressive numbers of users: In the third quarter of 2020, Facebook alone had 2.7 billion active monthly users (Statista, 2020c), and the Chinese WeChat more than 1.2 billion (Statista, 2020d). According to Our World in Data, “social media platforms are used by one-in-three people in the world, and more than two-thirds of all Internet users” (Ortiz-Ospina, 2019, para. 2). Online, one can broadcast a message to audiences of millions, whereas in face-to-face communication, there are physical limits to how many people can join a conversation (Barasch & Berger, 2014). Yet even though social media enables people to establish larger social networks and profit from greater global connectivity, the structures of social circles online and the number of close friends people have online do not significantly differ from their offline counterparts (Dunbar et al., 2015).^6^ In online social networks, the average number of friends (between 100 and 200) as well as the number of friends who are considered to belong to the two closest circles (typically around five and 15, respectively) do not differ from the values for offline inner circles (Dunbar, 2016; Dunbar et al., 2015). This suggests that the cognitive and temporal constraints that “limit face-to-face networks are not fully circumvented by online environments” (Dunbar, 2016, p. 7).

#### Amount of information, limitless space and storage

Digital environments are not subject to the same constraints on information proliferation and storage found in physical surroundings. Online space is virtually limitless, contains several layers (e.g., surface Web and dark Web), and can grow at a high pace. Consider that when Sergey Brin and Larry Page launched Google in 1998, they archived 25 million individual pages. In 2013 that number had grown to 30 trillion and, by 2016, had reached more than 130 trillion (Schwartz, 2016). At the time of this writing in the second quarter of 2020, there were 1.8 billion websites on the Internet and approximately 4.5 billion Google searches a day.^7^ Moreover, the potential for speed and scope of information propagation is much higher online, where the same message can be effortlessly and immediately copied to reach vast audiences. For example, the most shared tweet to date^8^ reached 4.5 million retweets, most of which happened in the 24 hr after the initial posting. New technologies have systems made for processing and storing information superior to any previously available systems (Clowes, 2013). This feature of digital technology also implies that information does not have an expiration date and can be stored more or less indefinitely—a situation that prompted the EU to establish what is commonly referred to as the “right to be forgotten,” which provides European citizens with a legal mechanism for ordering the removal of their personal data from online databases (European Parliament, 2016, Article 17).

#### Rapid change and adaptivity

Digital environments develop at a high rate, especially compared with most offline environments. The document-based Web 1.0 was replaced by the more interactive Web 2.0 in the beginning of the 2000s, and an increasingly more sophisticated and AI-powered Web of data is being introduced (Aghaei et al., 2012; Fuchs et al., 2010). Online content can be added, removed, or changed in seconds, and digital architectures can rapidly adapt to new demands and challenges. Even small changes in the structure of online architectures can have major societal consequences: For example, introducing some friction into the process of sharing information (i.e., increasing the investment in time, effort, or money required to access or spread information) can significantly decrease the likelihood of citizens engaging with the affected sources, as the Chinese government’s attempt to manage and censor information shows (see Roberts, 2018). Clicks, likes, and other types of social information shared online—as insignificant as they may seem individually—can collectively amount to sizable changes (e.g., for election results, which can in some cases be decided by just a few votes). For example, in a large-scale experiment on their users’ newsfeeds, Facebook showed that including social information in an “I voted” button (in this case, displaying faces of friends who had clicked on the button) affected both click rates and real-world voting—people who saw social information were 2.08% more likely to click on the button compared with those who saw nonsocial information, and they were 0.39% more likely to vote than were people who saw an informational message or no message at all—suggesting that social signals from friends on social networks (especially close friends) contributed to the overall effect of the message on people’s voting behavior (Bond et al., 2012).

#### Intelligence, personalization, and datafication

The latest developments in the evolution of the Internet increasingly depend on datafication (the transformation of many aspects of the world and people’s lives into data^9^) and mediation of content by algorithms and other intelligent technologies (we expand on this in the AI-Assisted Information Architectures section). Increasing datafication leads to increasing surveillance and control over people’s information diets (Zuboff, 2019), and rapidly developing machine-intelligence technology spurs a gradual relinquishing of both public control and transparency surrounding the technology. For example, search engines and recommender systems (e.g., video suggestions on YouTube) routinely rely on machine-learning systems that outperform humans in many respects (e.g., RankBrain in Google). Such algorithms are both complex and nontransparent—sometimes for designers and users alike (Burrell, 2016). The opacity of machine-learning algorithms stems from their autonomous and self-learning character: They are given input and produce output, but the exact processes that generate these outputs are hard to interpret. This has led some to describe these algorithms as “black boxes” (Rahwan et al., 2019; Voosen, 2017). Modern-day online environments, unlike their offline counterparts, possess autonomous intelligence—be it purely domain-specific machine intelligence, crowdsourced human intelligence, or a powerful combination of both.

#### Choice architectures and the power of design

Another feature that distinguishes online environments from physical surroundings is the ubiquity and the power of the design that mediates people’s online experience. The design of an interface in which people encounter the complexity of interconnected information online—the “human interface” (Berners-Lee et al., 1992)—presupposes that it has a decisive role in how people perceive the information presented. In other words, there is no Internet without ubiquitous *choice architectures* (for a definition, see Table 1) that constrain, enable, and steer user behavior. The very nature of online platforms affords quick design of choice architecture: It might take years to make a city bike-friendly (e.g., by building new bike lanes), but adjusting default settings on online pages or introducing friction into the process of information sharing can take less than a day. However, the same flexibility and adaptability of online choice architectures that can be used by benevolent choice architects to promote positive behavior can also be used by commercial and ill-meaning actors (more on this in the Persuasive and Manipulative Choice Architectures section).

### Differences in perception and behavior

Differences between online and offline environments are to be found not only in their structural characteristics and functionality but also in people’s perception of them and the way their behavior might change online in light of these perceptions.

#### Social cues and communication

Online communication differs from face-to-face communication in several ways, including the potential for anonymity and asynchronicity, the ability to broadcast to multiple audiences, and the availability of audience feedback (Misoch, 2015). Another characteristic of online communication that was emphasized in early research into Internet communication concerns the lack of nonverbal or physical cues—such as body language or vocal expressivity—that are important for conveying and understanding emotion in face-to-face communication. This raised concerns that increased use of computer-mediated communication would lead to impoverished social interaction (the reduced-social-cues model; e.g., Kiesler et al., 1984). However, it has now been recognized that users adapt to the medium and substitute the lack of nonverbal cues in digital communication with other verbal cues, thereby achieving equal levels of affective content (Walther et al., 2005, 2015). Online environments also contribute to the development of social cues, offering additional nonverbal cues such as emoticons, “likes,” and shares to enrich online communication. However, social cues can mean different things to users and platforms: To a user, a “like” button signifies appreciation or attention; to a tech firm, it is a useful data point. In addition, digital social cues can leak more information—and more sensitive information—than people intend to share (e.g., sexual orientation, personality traits, political views), including information that can be exploited to psychologically target and manipulate users (Kosinski et al., 2013; Matz et al., 2017).

#### Reliability of information and cues for epistemic quality

Information available online often lacks not only the typical social cues found in face-to-face interaction but also the cues to its epistemic quality that are generally available offline, such as an indication of sources or authorship. One reason for this is that the Internet—“an environment of information abundance”—is no longer subject to traditional filtering through professional gatekeepers (Metzger & Flanagin, 2015, p. 447). Modern-day digital media replaces expert gatekeeping with either crowdsourced gatekeeping (e.g., Wikipedia) or automated gatekeeping (e.g., algorithms on social media; Tufekci, 2015). Although some online platforms deliberately construct information ecosystems that favor indicators of quality (e.g., references to sources, fact-checking) and have rules for content creation (e.g., Logan et al., 2010), much of the content shared on social networks and online blogs does not give users sufficient cues to judge its reliability. Allcott and Gentzkow (2017) pointed out that because of the low costs of producing content, information online can be relayed among users with no significant third party filtering, fact-checking, or editorial judgment. An individual user with no track record or reputation can in some cases reach as many readers as Fox News, CNN, or *The New York Times* (p. 211).

Moreover, social-media platforms contribute to the phenomenon of “snack news”—“a news format that briefly addresses a news topic with no more than a headline, a short teaser, and a picture” (Schäfer, 2020, p. 1). Schäfer (2020) argued that frequent exposure to snack news can indirectly lead to the formation of strong convictions that are based on an illusory feeling of being informed. This phenomenon is not rare; an analysis by Bakshy et al. (2015) of 10.1 million Facebook profiles showed that users follow the links of only 7% of the news posts that appear in their news feeds. Moreover, manipulative use of certain cues—for instance, creating fake-news websites, impersonating well-known sources and social-media accounts, inflating emotional content (Crockett, 2017), or creating an illusion of consensus (Yousif et al., 2019)—can lead to dubious or outright false claims and ideas being disseminated.

Unreliable and intentionally fabricated false information is not found only in the digital world. Deception such as lying or impersonation is common offline as well (e.g., Gerlach et al., 2019). But because of some of the structural features we have discussed here (e.g., group size), deceptive acts can reach a much larger audience online than in face-to-face interactions. Impersonating an individual is easier when the important and hard-to-fake cues that are normally used to verify a person’s identity (e.g., voice, appearance, behavior) are not readily available. The perception (accurate or inaccurate) that false information and deception is more prevalent in the online world may exact far-reaching consequences. For instance, Gächter and Schulz (2016) recently showed, using cross-societal experiments from 23 countries, that the prevalence of rule violations across societies may impair individual intrinsic honesty, which is crucial for the functioning of a society.

#### Social calibration

The Internet can also affect social calibration—that is, perceptions about the prevalence of opinions in the general population. Offline, one gathers information about how others think from the limited number of people with whom one interacts, and most of these people live nearby. In the online world, physical boundaries cease to matter; one can connect with people around the world. One consequence of this global connectivity is that small minorities of people can form a seemingly large, if dispersed, community online. This in turn can create the illusion that even extreme opinions are widespread—thereby contributing to “majority illusion” (Lerman et al., 2016) and “false consensus” effects (the perception of one’s views as relatively common and of opposite views as uncommon; Leviston et al., 2013; Ross et al., 1977). Although one may find it very difficult to meet people in real life who believe the Earth is flat, the same is not true online among Facebook’s billions of users, where indeed there are some who do share this belief—or other equally exotic ones—and they can now find and connect with each other. Still another consequence of global connectivity is that online social media “dramatically increase the amount of social information we receive and the rapidity with which we receive it, giving social effects an extra edge over other sources of knowledge” (O’Connor & Weatherall, 2019, p. 16).

#### Self-disclosure and privacy behavior

The emergence and development of new online environments has consequences not only for how people communicate with others or how they evaluate information but also for the way they disclose information about themselves. Early studies on self-disclosure (revealing personal information to others) reported higher levels of sharing in visually anonymous computer-mediated communication than in face-to-face communication (Joinson, 2001; Tidwell & Walther, 2002). People also tend to be more willing to disclose sensitive information in online surveys that have the reduced social presence of the surveyor (Joinson, 2007). A systematic literature review by Nguyen et al. (2012) reported mixed evidence: Although most experimental studies (four of six) that measured self-disclosure showed more disclosure in online than in face-to-face interactions, in survey studies, participants reported more disclosure and willingness to share information with their offline friends (six of nine surveys). One may speculate that although the level of closeness, trust, and depth of interactions may prompt people to disclose personal information in offline relationships, the anonymity afforded by online communication can enhance people’s willingness to share. The benefits of online anonymity include the elimination of hierarchical markers (e.g., gender and ethnicity) that may trigger hostility (Young, 2002) and a sense of control people have over the information they share that stems from a belief that it will not be linked to their real personas. However, this sense of control can backfire. For example, one study showed that increasing individuals’ perceived control over the release and access of private information can increase their willingness to disclose sensitive information (the control paradox; Brandimarte et al., 2013).

Another paradox in people’s privacy behavior online is the *privacy paradox*: On one hand, people claim to care a great deal about their online privacy, but on the other, they appear to show little concern for it in their actual behavior (e.g., Acquisti et al., 2015; Norberg et al., 2007; for reviews, see Barth & De Jong, 2017; Kokolakis, 2017). However, a meta-analysis by Baruh et al. (2017) demonstrated that privacy concerns predict the extent to which individuals engage in privacy management (even though it does not eliminate the discrepancy). To the extent that the discrepancy between concerns and behavior does exist, it is possible that it reflects how people trade off immediate advantages of service use versus future risks toward their data privacy (which is one of possible explanations of the privacy paradox; see, e.g., Acquisti et al., 2015; Barth & De Jong, 2017). Another likely reason for this discrepancy between what people say about online privacy and what they actually do is the lack of transparency and associated lack of understanding of how online platforms collect and use people’s data and what can be inferred from that data. For instance, according to a survey by the Pew Research Center, 74% of Americans did not know that Facebook maintained a list of their interests and traits (Hitlin & Rainie, 2019).

#### Norms of civility

The “online disinhibition effect” describes “a lowering of behavioral inhibitions in the online environment” (Lapidot-Lefler & Barak, 2012, p. 434) that is not seen offline. Online disinhibition can be both benign and toxic (Suler, 2004): It can inspire acts of generosity and help shy people socialize, but it can also lead to increased incivility in online conversations—as behavior “that can range from aggressive commenting in threads, incensed discussion and rude critiques, to outrageous claims, hate speech, and more severe forms of harassment such as purposeful embarrassment and physical threats” (Antoci et al., 2019, p. 84). One of the most common examples of incivility is trolling, a type of online harassment that involves “posting inflammatory malicious messages in online comment sections to deliberately provoke, disrupt, and upset others” (Craker & March, 2016, p. 79). Trolling can be used strategically to disrupt the possibility of constructive conversation. Incivility is pervasive online: A survey by the Pew Research Center revealed that 44% of Americans have personally experienced online harassment, and 66% have witnessed it being directed at others (Duggan, 2017). Although incivility in online comments can polarize how people perceive issues in the media (A. A. Anderson et al., 2014) and can disproportionally affect female politicians and public figures (Rheault et al., 2019) and members of minority groups (Gardiner, 2018), it seems to be perceived as the norm, rather than the exception, for online interaction (Antoci et al., 2019). One may speculate that actions in the online sphere might be perceived as less influential: For instance, insulting and even threatening anonymous users in online forums may be perceived as less harmful and consequential—for both the victim and the perpetrator—than threatening someone face to face.

#### Perception of reality

In contrast to the offline world, the Internet and social media are immaterial, virtual environments that do not exist outside of the human-created technology that supports them (McFarland & Ployhart, 2015). This relative lack of anchoring in the material world allows for multiple realities to be constructed for, or by, different audiences and media online (Waltzman, 2017), so that any reference to the objective truth and shared reality can be replaced by alternative narratives (e.g., “systemic lies” created to promote a hidden agenda; McCright & Dunlap, 2017). The impact of the Internet on the media landscape—along with several other factors, such as rising economic inequality and growing polarization—is likely to have contributed to the emergence of the “posttruth” environment, an alternative epistemic space “that has abandoned conventional criteria of evidence, internal consistency, and fact-seeking” (Lewandowsky, Ecker, & Cook, 2017, p. 360). In this alternative posttruth reality, deliberate falsehoods can be described as “alternative facts,” and politicians and media figures (on both sides of the Atlantic) can claim that objectivity “is a myth that is proposed and imposed on us” (Dmitry Kiselev, as quoted by Yaffa, 2014, para. 8), that “there’s no such thing, unfortunately, anymore as facts” (Scottie Nell Hughes, as quoted by Holmes, 2016, para. 3), or that “truth isn’t truth” (Rudy Giuliani, as quoted by Pilkington, 2018, para. 2; see also Lewandowsky, 2020b; Lewandowsky & Lynam, 2018). These environments are conducive to the dissemination of false news and rumors, which in turn undermine public trust in any information and erode the basis of shared reality (Watts & Rothschild, 2017), thereby creating an atmosphere of doubt that serves as a fertile ground for conspiracy theories (more on this in the False and Misleading Information section).

**Fig. 2.**
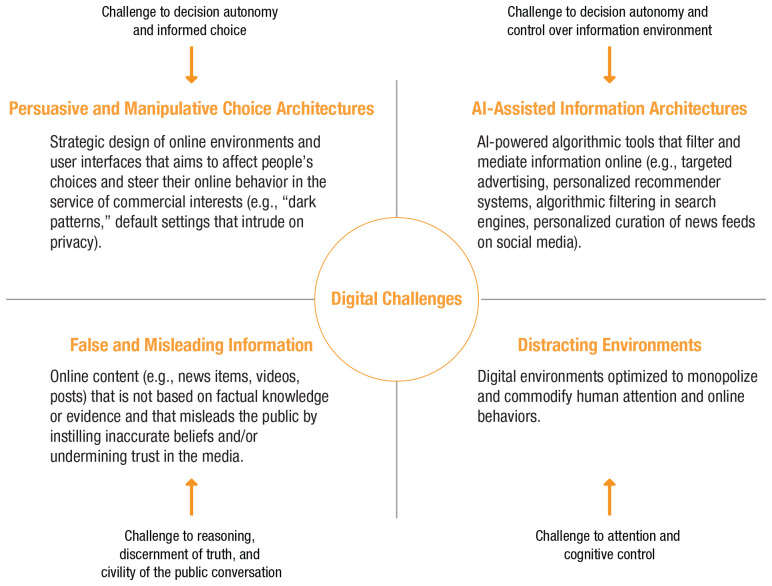
Challenges in the digital world.

To summarize, online and offline worlds differ in psychologically and functionally relevant ways. The online world appears to trigger perceptions that can render it different from the offline world. When people and online architectures are brought into contact (without much public oversight and democratic governance), pressure points will emerge. We next review four such challenges (outlined in Fig. 2): persuasive and manipulative choice architectures, AI-assisted information architectures, the proliferation of false and misleading information, and distracting environments.

## Challenges in Online Environments

### Persuasive and manipulative choice architectures

Modern online environments are replete with smart, persuasive choice architectures that are designed primarily to maximize financial return for the platforms, capture and sustain users’ attention, monetize user data, and predict and influence future behavior (Zuboff, 2019). For example, Facebook’s business model relies on exploiting user data to the benefit of advertisers; the goal is to maximize the likelihood that an ad captures its target’s attention. To stretch the time people spend on the platform (thus producing behavioral data and watching ads), Facebook employs a variety of design techniques that aim to change users’ attitudes and behavior via persuasive choice and information architectures (e.g., Eyal, 2014; Fogg, 2003). It is no coincidence that notifications are red; the color incites a sense of urgency. The “like” button triggers a quick sense of social affirmation. The bottomless news feed, with no structural stop to scrolling (i.e., infinite scroll), prompts people to consume more without noticing. These examples illustrate that a witting or unwitting awareness (via massive A/B testing) of human psychology underlies persuasive choice architectures and commercial nudging. Benefiting from an abundance of data on human behavior, these architectures are continuously being adapted to offer ever-more-appealing user interfaces to compete for human attention (e.g., Harris, 2016).

The main ethical ambiguity of persuasive choice architectures and commercial nudging resides in their close ties to other types of influence, such as coercion and, in particular, manipulation. Coercion is a type of influence that does not convince its targets but rather compels them by eliminating all options except for one (e.g., take-it-or-leave-it choices). Manipulation is a hidden influence that attempts to interfere with people’s decision-making processes to steer them toward the manipulator’s ends. It neither persuades people nor deprives them of their options; instead, it exploits their vulnerabilities and cognitive shortcomings (Susser et al., 2019). Manipulation thus undermines both people’s control and their autonomy over their decisions—that is, their sense of authorship and their ability to identify with the motives of their choices (e.g., Dworkin, 1988). It also prevents people from choosing their own goals and pursuing their own interests. Not all persuasive choice architectures are manipulative—only those that exploit people’s vulnerabilities in a nontransparent, covert manner. Below we consider two cases in which persuasive design in online environments borders on manipulation: dark patterns and hidden privacy defaults.

**Fig. 3.**
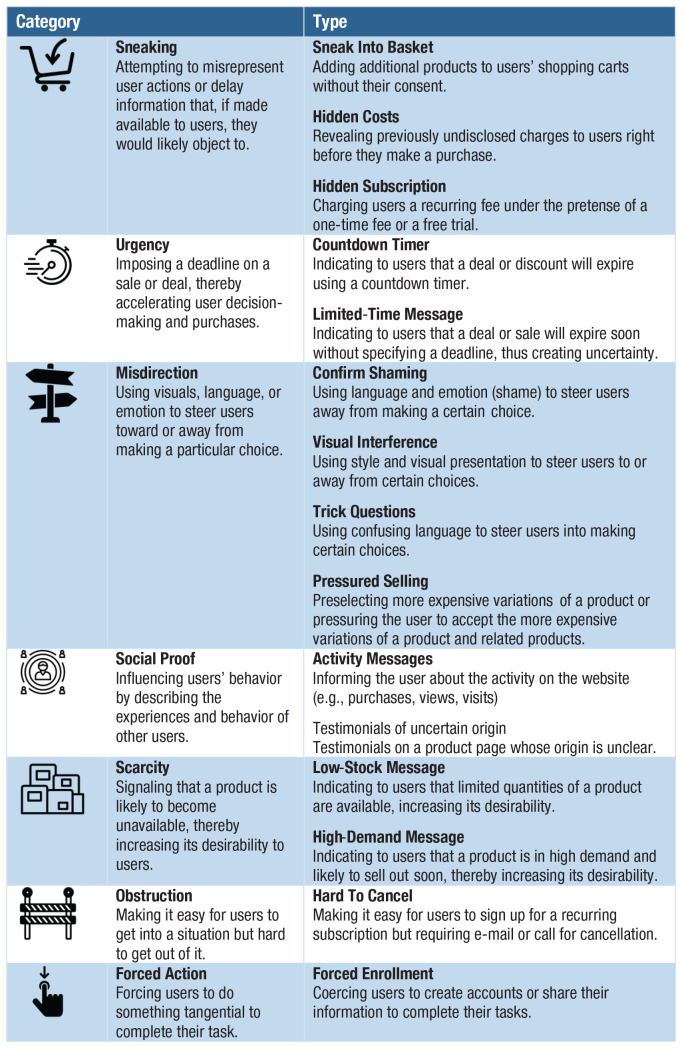
Categories and types of dark patterns. Source and visual materials: Dark Patterns Project at Princeton University (https://webtransparency.cs.princeton.edu/dark-patterns); see also Mathur et al. (2019). The icons are used with permission of the Dark Patterns Project.

Dark patterns—a term coined by designer and user-experience researcher Harry Brignull (see Brignull, 2019; Gray et al., 2018; Mathur et al., 2019)—are a manipulative and ethically questionable use of persuasive online architectures. “Dark patterns are user interface design choices that benefit an online service by coercing, steering, or deceiving users into making unintended and potentially harmful decisions” (Mathur et al., 2019, p. 1). One notorious example of dark patterns is the “roach motel,” unglamorously named after devices used to trap cockroaches. The roach motel makes it easy for users to get into a certain situation, but difficult to get out (in Fig. 3 it falls under the type “hard to cancel”). Many online subscription services function that way. For instance, creating an Amazon account requires just a few clicks, but deleting it is difficult and time consuming: The user must first hunt for the hidden option of deleting an account, then request this procedure by writing to customer service. This asymmetry in the ease of getting in and out borders on manipulation and retains customers. Another example is “forced continuity”: subscriptions that, after an initial free trial period, continue on a paid basis without notifying users in advance and without giving them an easy way to cancel the service.^10^

Dark patterns are anything but rare. In a recent large-scale study, Mathur et al. (2019) tested automated techniques that identified dark patterns on a sizeable set of websites. They discovered 1,818 instances of dark patterns from 1,254 websites in the data set of 11,000 shopping websites. Mathur et al.’s findings revealed 15 types of dark patterns belonging to seven broader categories (see Fig. 3), such as misdirection, applying social pressure, sneaking items into the user’s shopping basket, and inciting a sense of urgency or scarcity (a strategy often used by hotel-booking sites or airline companies).

Another case of persuasive design that borders on manipulation is hidden default settings. Hidden defaults present a particularly strong challenge because they trick people into accepting settings without being fully (if at all) aware of the consequences. For example, online platforms are often designed to make it difficult to discontinue personalized advertising or choose privacy-friendly settings. Default settings can also lead users to unwittingly share sensitive data, including location information that can be used to infer attributes such as income or ethnicity (see, e.g., Lapowsky, 2019). Default data-privacy settings do not even have to follow dark-patterns strategies: Most users, lacking the time or motivation to go several clicks deep into the settings labyrinth, will not change their defaults unless they have a specific reason to do so. Hidden defaults raise clear ethical concerns, but these practices continue despite the introduction of the GDPR in Europe in 2016, which stresses the importance of privacy-respecting defaults and insists on a high level of data protection that does not require users to actively opt out of the collection and processing of their personal data (European Parliament, 2016, Article 25).

However, attempts to game the rules of informed consent and privacy by default have found to be a major challenge to GDPR implementation. Nouwens et al. (2020) reported that dark patterns and hidden defaults in the form of implied consent are ubiquitous on new consent-management platforms (in the United Kingdom) and that only 11.8% meet minimal requirements of GDPR for valid consent (e.g., no prechecked boxes, explicit consent, rejecting as easy as accepting). According to a report by the Norwegian Consumer Council (2018), tech companies such as Google, Facebook, and—to a lesser extent—Microsoft use design choices in “arguably an unethical attempt to push consumers toward choices that benefit the service provider” (p. 4). On the topic of privacy, key findings of the report include the use of privacy-intrusive default settings (e.g., Google requires that the user actively go to the privacy dashboard to disable personalized advertising), framing and wording that nudges users toward a choice by presenting the alternative as ethically questionable or highly risky (e.g., on Facebook: “If you keep face recognition turned off, we won’t be able to use this technology if a stranger uses your photo to impersonate you”), giving users the illusion of control (e.g., Facebook allows users to control whether Facebook uses data from partners to show them ads, but not whether the data are collected and shared in the first place), take-it-or-leave-it choices (e.g., a choice between accepting the privacy terms or deleting an account), and design of choice architectures in which choosing the privacy-friendly option requires more effort from the users (Norwegian Consumer Council, 2018). Such design choices might also contribute to the privacy paradox by actively discouraging users from behaving in a way that reflects their concern for their privacy. Users’ halfhearted privacy-protecting behavior might be due not to laziness or a lack of skills but rather to the unnecessarily complicated nature of protecting one’s privacy online.

In sum, persuasive designs and commercial nudges can go far beyond transparent persuasion and enter the territory of hidden manipulation when they rely on dark patterns (Mathur et al., 2019), default settings that intrude on user privacy (Norwegian Consumer Council, 2018), and the exploitation of people’s biases and vulnerabilities (Susser et al., 2019). These practices affect not only how users access information but also what information they agree to share. Moreover, online manipulation undermines people’s control and autonomy over their decisions by nudging them toward behaviors that benefit commercial actors or by hiding relevant information (e.g., settings for discontinuing personalized advertisement).

### AI-assisted information architectures

**Fig. 4.**
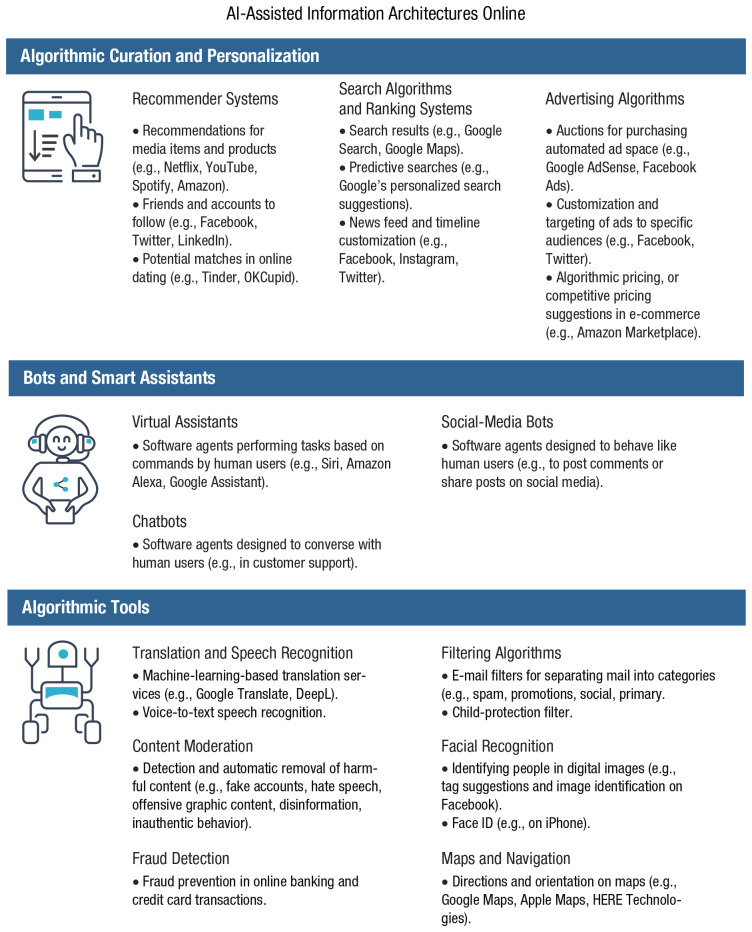
Examples of AI-assisted information architectures online. Icons are used under license from Adobe Stock.

Another challenge of online information and choice architectures comes with the use of machine learning and smart algorithms. We use the term *AI-assisted information architectures* to describe a variety of AI-powered algorithmic tools that filter and mediate information online. These tools include personalized targeted advertising, personalized recommender systems, algorithmic filtering in search engines, and customized news feeds on social media (for an overview, see Fig. 4). Algorithmic filtering and personalization are not inherently malicious technologies—on the contrary, they are helpful tools that allow people to navigate the overwhelming amount of information on the Internet. Instead of showing countless random results for search queries, search engines aim to offer the most relevant results. For a user in Sydney, Australia, Googling “Newcastle” should prioritize information about the city that is 200 km to the north, not its distant British namesake. In a similar vein, news feeds on social media strive to show news that is interesting to users. Recommender systems offer content suggestions on the basis of users’ past preferences and the preferences of users who are inferred to have similar tastes (e.g., video suggestions on Netflix and YouTube). Besides selecting information on the basis of its personalized relevance, algorithms can also filter out information that is considered to be harmful or unwanted, for instance by automatically filtering spam or removing hate speech and disturbing videos (the majority of hate speech on Facebook is removed by its machine-learning algorithms; see Chart 1 in “Social media’s struggle with self-censorship,” 2020). There are countless examples of why filtering information on the Internet is indispensable and helpful and why automation makes this daunting process more efficient (e.g., Rainie & Anderson, 2017), and there are many ways in which algorithms can support human decision-making (Christian & Griffiths, 2016). Automated algorithmic systems act as buffers between the abundance of information and the scarcity of human attention. However, they are not without some notable problems.

One general problem is that decision-making is being delegated to a variety of algorithmic tools without clear oversight, regulation, or understanding of the mechanisms underlying the resulting decisions. For example, ranking algorithms and recommender systems are considered proprietary information, and therefore neither individual users nor society in general has a clear understanding of why information in search engines or social-media feeds is ordered in a particular way (Pasquale, 2015). Other factors contribute further to the lack of transparency,^11^ such as the inherent opacity of machine-learning algorithms (the black-box problem) and the complexity of algorithmic decision-making processes (de Laat, 2018; Turilli & Floridi, 2009). Delegating decision-making this way not only results in impenetrable algorithmic decision-making processes but also precipitates people’s gradual loss of control over their personal information and a related decline in human agency and autonomy (J. Anderson & Rainie, 2018; Mittelstadt et al., 2016; Zarsky, 2016). Relatedly, data privacy and its protection in the context of AI-assisted information environments should be seen not merely as an individual good but as a public good (Fairfield & Engel, 2015). As algorithmic inferences from data collected from users can be used to predict personal information of nonusers (known as *shadow profiles*; see Garcia, 2017), privacy may be at risk not because of an individual’s own actions but because others have been unconcerned about the privacy of their data or because online choice architectures have “nudged” others toward privacy-unfriendly options (e.g., Utz et al., 2019).

Consistent delegation of choice and shifting autonomy from users to algorithms leaves open the question of responsibility and accountability (Diakopoulos, 2015). Because artificial agents are capable of making their own decisions and because no one has decisive control over their actions, it is difficult to assign responsibility for the outcomes (e.g., the responsibility gap; see Matthias, 2004). Consider the decisions of a recommender system employed on YouTube (boasting about 2 billion users, it is the second most popular social network and the second most visited website worldwide^12^). The recommender algorithm—based on deep neural-network architecture—offers video recommendations to YouTube users with the predominant purpose of increasing watching time (Covington, Adams, & Sargin, 2016). However, one unintended consequence happened to be that the system promoted videos that tended to radicalize their viewers with every step. For example, Tufekci (2018) reported how after showing videos of Donald Trump during the 2016 presidential campaign, YouTube started to recommend and autoplay videos featuring White supremacists and Holocaust denialists. After playing videos of Bernie Sanders, YouTube suggested videos on left-wing conspiracies (e.g., that the U.S. government was behind the 9/11 attacks). An investigation by *The Guardian* in cooperation with the former Google engineer Guillaume Chaslot demonstrated the biased nature of YouTube recommendations and stated that it “systematically amplifies videos that are divisive, sensational and conspiratorial” (Lewis, 2018, para. 25; see also Lewis & McCormick, 2018). There is now evidence suggesting that these algorithms may have actively contributed to the rise and unification of right-wing extremists in the United States (Kaiser & Rauchfleisch, 2018), Germany (Rauchfleisch & Kaiser, 2017), and Brazil (Fisher & Taub, 2019). It is unlikely that these are the only affected countries or that YouTube is the only platform with this problem. For instance, Facebook recommendation tools (“Groups you should join” and “Discover” algorithms), according to the company’s own internal report, have been implicated in the growth of extremists groups on the platform (see Horwitz & Seetharaman, 2020).

Who, then, should be held accountable for decisions made by autonomous recommender systems that suggest ever more radical content on social networks: the developers of the algorithms, the owners of the platforms, or the content creators? YouTube recently vowed to limit recommending conspiracy theories on its platform (Wong & Levin, 2019), a move that highlights the tech industry’s unilateral power to shape their users’ information diets. In a recent empirical audit of YouTube recommendations, Hussein, Juneja, and Mitra (2020) found that the YouTube approach indeed limited recommendations of selected conspiracy theories (e.g., the flat-earth narrative) or medical misinformation (videos promoting vaccine hesitancy), but not of other misinformation topics (e.g., the chemtrail conspiracy narrative).

Another closely related concern is the impact of AI-driven algorithms on choice architectures—for instance, when algorithms function as gatekeepers, deciding what information should be presented and in what order (Tufekci, 2015). Be it personalized advertising or filtering information to present the most relevant items, the results directly affect people’s choices by narrowing their options (Newell & Marabelli, 2015) and steering their decisions in a particular direction or reinforcing existing attitudes (e.g., Le et al., 2019). The consequences loom large for societies as a whole as well as for individuals: Epstein and Robertson (2015) showed, using a simulated search engine, that rankings favoring a particular political candidate can shift voting preferences of undecided voters by 20% or more. Given that four of the past five U.S. presidential elections resulted in margins between the Democrats and Republican of below 4% and that the 2016 election, for instance, was decided by razor-thin margins in a few swing states (six states were won by margins of less than 2%), the impact of potential search-engine biases should not be ignored.

Microtargeted advertisement on social media, especially in the context of political campaigning, is another case in point. This method relies on automated targeting of messages on the basis of people’s personal characteristics (as extracted from their digital footprints) and a use of private information that stretches the notion of informed consent (e.g., psychographic profiling; see Matz et al., 2017). The resulting microtargeted political messages, which are seen only by the targeted audience, can exploit people’s psychological vulnerabilities while evading public oversight. Findings show that data collected about people online can be used to make surprisingly accurate inferences about people’s sexual orientation, personality traits, and political views (Kosinski et al., 2013). For instance, algorithmic judgments about people’s personalities that are based on information extracted from digital fingerprints (e.g., Facebook likes) can be more accurate than judgments made by relatives and friends (Youyou et al., 2015), and just 300 likes are sufficient for an algorithm to predict users’ personalities more accurately than their own spouses can (Youyou et al., 2015). In a systematic review of 327 articles, Hinds and Joinson (2018) showed that multiple pieces of demographic data could be reliably inferred from people’s digital footprints, including ethnicity, occupation, and sexual orientation. Hinds and Joinson (2019) also demonstrated that computer-based predictions of personality traits (e.g., extraversion, neuroticism) from digital footprints are more accurate than human judges’ predictions. This information can be used to create a dangerous “personality panorama” (Boyd et al., 2020) of people’s behavior online that, consequently, can be employed to persuade and manipulate users; for example, advertising messages can be adjusted to match people’s introversion or extroversion score (Matz et al., 2017). A former employee reported that Cambridge Analytica used personality profiling during Donald Trump’s 2016 presidential campaign to target fear-based messages (e.g., “Keep the terrorists out! Secure our borders!”) to people who scored high on neuroticism (Amer & Noujaim, 2019).

The impact of this manipulation on the outcomes of the Brexit vote and the 2016 U.S. election is a major cause for concern and an argument for stricter regulation of online platforms (e.g., Jamieson, 2018; Persily, 2017; Digital, Culture, Media and Sport Committee, 2019). Sixty-two percent of social-media users in the United States agree that it is not acceptable for social-media platforms to use their data to deliver customized messages from political campaigns (Smith, 2018b). Recent surveys in Germany, the United Kingdom, and the United States (Ipsos MORI, 2020; Kozyreva et al., 2020) also provide evidence that people consider personalization and targeting in political campaigning unacceptable. The impact of microtargeting is often exacerbated by the lack of transparency in political campaigning on social media: It is nearly impossible to trace how much has been spent on microtargeting and what content has been shown (e.g., Dommett & Power, 2019).

Another challenging consequence of algorithmic filtering is algorithmic bias (e.g., Bozdag, 2013; Corbett-Davies et al., 2017; Fry, 2018). Here ethical concerns touch on both the generation of biases in data processing and the societal consequences—such as discrimination—of implementing biased algorithmic decisions (Mittelstadt et al., 2016; Rahwan et al., 2019). One particularly disturbing set of examples concerns deeply rooted gender or racial biases that can be picked up by data-processing algorithms. One study of personalized Google advertisements demonstrated that setting the gender to female (rather than male) in simulated user accounts resulted in fewer ads related to high-paying jobs (Datta et al., 2015). Another study found that online searches for “Black-identifying” names were more likely to be associated with advertisements suggestive of arrest records (e.g., “Looking for Latanya Sweeney? Check Latanya Sweeney’s arrests”; Sweeney, 2013, p. 3). Names such as Jill or Kristen did not elicit similar ads even when arrest records existed for people with those names. Striking examples of racial biases in algorithmic decision-making are not limited to online environments; they also have consequential effects offline, for instance in policing and health (e.g., Obermeyer et al., 2019).

Algorithms are designed by human beings, and they rely on existing data generated by human beings. They are therefore likely not only to generate biases because of technical limitations but also to reinforce existing biases and beliefs (Bozdag, 2013), which in turn can deepen ideological divides and exacerbate political polarization. Along the same lines, it has been argued that personalized filtering on social-media platforms may be instrumental in creating “filter bubbles” (Pariser, 2011) or “echo chambers” (Sunstein, 2017); echo chambers are information environments “in which individuals are exposed only to information from like-minded individuals” (Bakshy et al., 2015, p. 1130), whereas filter bubbles refer to content selection “by algorithms according to a viewer’s previous behaviors” (p. 1130). Both echo chambers and filter bubbles tend to amplify the confirmation bias—a way to search for and interpret information that reinforces preexisting beliefs and increases political polarization (e.g., Bail et al., 2018) and radicalization. Not everyone agrees about the existence of filter bubbles, however; some researchers argue that news-audience fragmentation is less prevalent than is often assumed (Flaxman et al., 2016) or that face-to-face interaction is currently even more segregated than online discourse (Gentzkow & Shapiro, 2011). Bakshy et al. (2015) found that individual choices, not algorithms, limit exposure to attitude-challenging views among Facebook users. But because recommender systems typically “learn” users’ preferences, psychological tendencies in information selectivity and algorithmic amplification of those tendencies are likely to reinforce one another.

From a psychological perspective, many factors could motivate online segregation and polarization, including confirmation bias, selective exposure to information, and selective engagement with online content (e.g., Garrett & Stroud, 2014). Although exposure might be not as segregated as is commonly claimed, social-media environments show signs of selective engagement (in the form of likes, shares, and comments; see Schmidt et al., 2017), leading to “highly segmented interaction with social media content” (Garrett, 2017, p. 371). The extent to which such selective exposure and engagement can distort people’s information diets and influence democratic processes is highly debated—we return to this topic in the next section.

### False and misleading information

**Fig. 5.**
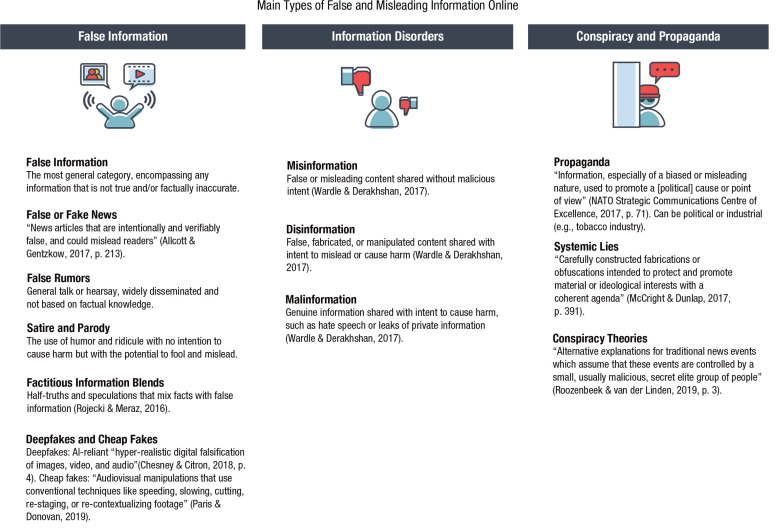
Main types of false and misleading information in the digital world. Icons are used under license from Adobe Stock.

Another challenge presented by online environments and social networks is the increasing speed and scope of false-information proliferation and its resulting threat to the rationality and civility of public discourse—and ultimately to the very functioning of democratic societies. In this section we explore three questions: (a) What is the extent of the “false news” problem? (b) What are useful taxonomies of false and misleading information? (c) What are the psychological mechanisms underlying receptivity to false content online? Before we proceed, let us briefly mention our terminological choices. We use the term *disinformation* to refer to false and misleading information spread with malicious intent, and we use *misinformation* for cases when the intent is unknown or irrelevant (as in Wardle & Derakhshan, 2017). We generally use the term *false news* (instead of fake news) to refer to inaccurate information presented as news. However, we make an exception when discussing results from scientific articles that use “fake news” to refer to, for instance, “false and misleading information masquerading as legitimate news” (Allen et al., 2020, para. 1). We address the limitations of the “fake news” terminology and other useful classifications, including mis- and disinformation, after discussing the scope of the problem (also in Fig. 5).

#### Scope of the problem

We begin our review of the challenge of false and misleading information with an examination of its scope. A recent report by Bradshaw and Howard (2019) showed that in the past 2 years alone, the number of countries with disinformation campaigns more than doubled (from 28 in 2017 to 70 in 2019), and that Facebook remains the main platform for those campaigns. At least half of all Internet users rely on online and social media as their primary sources of news and information, including 36% who access Facebook for news (Newman et al., 2020). False and unverified claims online can therefore lead not only to false beliefs and misguided actions but also to an erosion of trust in the information ecosystem—ultimately threatening a society’s ability to hold evidence-based conversations and reach a consensus. There is much concern that the spread of false news and rumors on Facebook and Twitter influenced the U.S. presidential election and the Brexit referendum in 2016 (see Digital, Culture, Media and Sport Committee, 2019; Persily, 2017). For instance, Allcott and Gentzkow (2017) estimated that the average U.S. adult read and remembered at least one fake-news article during the election period. They also compiled a database of fake-news articles that circulated in the 3 months before the 2016 election (115 pro-Trump and 41 pro-Clinton) and that, together, were shared 38 million times in the week leading up to the election. As Silverman’s (2016) analysis showed, in the 3 months before the 2016 U.S. presidential election, the most popular false-news stories were more widely shared on Facebook than the most popular mainstream news stories. The 20 top-performing false election stories from hoax sites and hyperpartisan blogs generated 8,711,000 shares, reactions, and comments, whereas the 20 top-performing election stories from legitimate news websites generated 7,367,000 reactions. A single false story about the Pope endorsing Donald Trump was liked or shared on Facebook 960,000 times.

Online disinformation and misleading claims can have deadly real-world consequences: The Pizzagate conspiracy theory, which alleged that Hillary Clinton and her top aides were running a child-trafficking ring out of a Washington pizzeria, was floated during the 2016 presidential campaign on Reddit, Twitter, and fake-news websites. It led to repeated harassment of the restaurant’s employees and eventually prompted an armed 28-year-old man to open fire inside the pizzeria (Aisch, Huang, & Kang, 2016). On a broader—and even more disturbing—scale, the Myanmar military orchestrated a propaganda campaign on Facebook that targeted the country’s Muslim Rohingya minority group, inciting violence that forced 700,000 people to flee (Mozur, 2018). Encrypted messenger networks such as WhatsApp are also vulnerable to manipulation: False rumors about child kidnappers shared in Indian WhatsApp groups in 2018 incited at least 16 mob lynchings, leading to the deaths of 29 innocent people (Dixit & Mac, 2018). Most recently, the COVID-19 pandemic has given rise to multiple conspiracy theories and misleading news stories that gain credibility among members of the public by exploiting their fears and uncertainty; for instance, 29% of Americans believe that COVID-19 was created in a lab (Schaeffer, 2020), and there have been up to 50 attacks on mobile-phone masts in the UK since the spread of coronavirus was fallaciously linked to the country’s rollout of the 5G mobile network (Adams, 2020).

Several recent analyses have suggested that the problem of fake news is not as serious as was initially believed in the aftermath of Brexit and the 2016 U.S. election (Allen et al., 2020; Grinberg et al., 2019; Guess et al., 2019; Guess, Lockett, et al., 2020; Guess, Nyhan, & Reifler, 2020). Table 3 summarizes these articles, which all used big-data analyses to measure Americans’ exposure to fake news and concluded that the limited prevalence of fake news online (of the type examined in these articles)^13^ may not present cause for alarm.

**Table 3.** Summary of Recent Big-Data Analyses of Fake News Consumption in the United States

Study	Sampling time	Source data	Results	Number of URLs
Allen et al. (2020)	Jan. 2016–Dec. 2018	TV consumption and all online consumption (mobile and desktop). Also “imputed passive consumption” (items that appear in feed but were not clicked on by user) for top four sites (Facebook, YouTube, Twitter, Reddit) and top three search engines (Google, Bing, Yahoo).	• TV consumption outweighs online consumption by factor of 5 • Fake news constitutes only 0.15% of daily media diet • For a very small number (0.7% of panel), more fake news was consumed than real news • Older people are more exposed to and spend more time with fake news	98^a^
Grinberg et al. (2019)	Aug. 2016–Dec. 2016	Twitter feed (URLs only) from a sample (*N* = 16,442) of registered voters.	• 5% of political exposure on Twitter was fake news • Average proportion of fake news in an individual’s feed was 1.18% • 1% of individuals accounted for 80% of fake news source exposures • 0.1% accounted for nearly 80% of fake news sources shared • Far more exposure and sharing on right than left and by older people than by younger people	171 + 64 + 65^b^
Guess, Nyhan, & Reifler (2020)	Oct. 2016–Nov. 2016	Web consumption of (demographically) representative online panel (*N* = 2,525), excluding Facebook news feed and mobiles.	• 5.9% of news articles during 2016 election were from untrustworthy sites • 44.3% of Americans visited untrustworthy sites • 62% of traffic to untrustworthy websites came from the 20% of news consumers with the most conservative information diets • Older people also consumed more • Access to untrustworthy sites increased with Facebook usage	382 + 61 + 47^c^
Guess et al. (2019)	Nov. 2016–Jan. 2017	Facebook sharing data combined with online survey from a representative panel (*N* = 1,191).	• More than 90% shared no stories from fake-news domains • 8.5% of respondents shared at least one fake-news article • Sharing vastly greater among conservatives and older people (people over 65 share 7 times as much as others) • Sharing of fake news not related to overall sharing quantity; not the case that “some people will share anything”	21^d^
Guess, Lockett, et al. (2020)	June 2018–Dec. 2018	Survey (*N* = 18,733 across two waves, each in three parts—summer, fall, and winter) with all web browsing data (limited access to mobile usage).	• Fake-news consumption associated with low trust in media and greater affective polarization. • Fake news associated with greater belief in pro-Republican misperceptions (even after controlling for partisanship) • Fake news not associated with political participation • Additional experimental results not reported here	171 + 64 + 65^b^

a The 98 URLs were truncated by traffic (eliminating low-traffic sites) from a full list of 642 sites formed by combining Grinberg et al. (2019)’s “red” and “black” sites with lists created by NewsGuard and Buzzfeed. ^b^Building on previous research, a list was compiled of 171 “black” sites (almost exclusively fabricated), 64 “red” sites (flawed editorial process), and 65 “orange” sites (less likely to be systematically flawed). Additional black sites were curated but did not appear in the data. ^c^Three hundred eighty-two “black” sites, 61 “red,” and 47 “orange” combined from Grinberg et al. (2019) and (Guess et al., 2019). ^d^List of fake news sites curated by a journalist (Silverman at Buzzfeed), reduced to 21 sites.

Although there is notable heterogeneity among the articles shown in Table 3, particularly in the source of data, the analyses identify at least three consistent attributes of the problem of fake news: First, the distribution of fake-news consumption and sharing is extremely lopsided; most people are not involved at all, and a small number of users are responsible for the lion’s share of consumption and sharing. Second, age appears to be an important variable: People over the age of 65 share far more fake news than do younger adults. Finally, the political distribution is highly asymmetrical. Although some fake news appeals to left-wing views, the majority of fake news is consonant with right-wing attitudes. Accordingly, people on the far right, and Trump supporters in particular, share considerably more fake news than do moderates or liberals.

The articles also share a methodological commonality that reveals a strong limitation: They all operationalize exposure to or sharing of fake news by counting visits to or shares of a limited number of specific websites (Table 3, final column). Fake-news outlets were defined as sites that have the trappings of legitimately produced news but lack the editorial standards or processes to ensure accuracy (Grinberg et al., 2019). Examples included conservativetribune.com, wnd.com, and rushlimbaugh.com. Lists of those sites were carefully curated by a variety of sources (Table 3, footnotes) and cross-checked against fact-checker performance (e.g., Guess, Nyhan, & Reifler, 2020). One can therefore state with confidence that those sites were purveyors of fake news. Most articles listed in Table 3 also showed that the authors’ conclusions were robust to extensions and alterations of their lists. Yet the articles did not consider any other forms of political material online as potential sources of fake news; false advertisements, unchecked false statements by politicians, and false or misleading information in mainstream media were not included in the analyses. Moreover, looking at click-through rates considerably underestimates exposure to false news because most people do not follow the link in the headlines that they see on their social-media feeds (see e.g., Bakshy et al., 2015).

The results in Table 3 therefore present a lower bound on exposure to false and misleading information online. Their converging suggestion that few people visit or share material from fake-news sites does not speak to the magnitude of the disinformation problem overall (as noted by Allen et al., 2020). Concern about the widespread effects of misinformation on society is therefore justified (e.g., Bradshaw & Howard, 2019; Roozenbeek & van der Linden, 2018; Zerback et al., 2020). These legitimate concerns are fueled by a number of issues. For example, Facebook has an explicit policy against fact-checking political advertisements (Wagner, 2020), which was—unsurprisingly—exploited during the December 2019 election in the UK. According to fact-checkers, 88% of Facebook ads posted by the Conservative party during a sampling period immediately before the election were misleading, compared with around 7% of those posted by the Labour party (Reid & Dotto, 2019). In addition, even a small dose of fake news can set agendas in “its ability to ‘push’ or ‘drive’ the popularity of issues in the broader online media ecosystem” (Vargo et al., 2018, p. 2043). Vargo et al. (2018) showed that although fake news did not dominate the media landscape from 2014 through 2016, it was intertwined with American partisan media (e.g., Fox News); each influenced the other’s agendas across a wide range of topics, including the economy, education, the environment, international relations, religion, taxes, and unemployment.^14^

Last but not least, people’s perceived exposure to misinformation and disinformation online is high:^15^ In the EU, “in every country, at least half of respondents [in the sample of 26,576] say they come across fake news at least once a week” (Directorate-General for Communication, 2018, p. 2). In the United States, “about nine-in-ten U.S adults (89%) say they often or sometimes come across made-up news intended to mislead the public, including 38% who do so often” (Mitchell et al., 2019, p. 15). Globally (across 40 countries), 56% of respondents are concerned about what is real or fake when it comes to online news, and almost four in 10 (37%) said they had come across a lot or a great deal of misinformation about COVID-19 on social media, such as Facebook and Twitter (Newman et al., 2020).

#### Taxonomies of false and misleading information

What is online false and misleading information? Clearly, it is not a single homogeneous entity. For instance, dangerously misleading online content might arise from deliberate attempts to manipulate public opinion or emerge as an unintended consequence of sharing unverified rumors and false news. Focusing on information falseness and the intent to mislead, Wardle and Derakhshan (2017) distinguished among three types of “information disorders”^16^: *misinformation* (false or misleading content created and initially shared without malicious intent), *disinformation* (false, fabricated, or manipulated content shared with intent to mislead or cause harm), and *malinformation* (genuine information shared with intent to cause harm—e.g., hate speech and leaks of private information).

Although this classification establishes some useful general distinctions, the landscape of online falsehoods and propaganda is much more complicated. For example, the difference in intent between misinformation and disinformation is often hard to establish, and the real consequences of both can be equally harmful. Both are therefore usually considered to be false information—or, if presented as news, false (or fake) news. Moreover, there are additional categories of misleading content, such as online political propaganda and “systemic lies” (McCright & Dunlap, 2017); the latter are created and curated by organized groups with vested interests (e.g., fossil-fuel companies denying climate science). Likewise, motivations for creating false content can be financial as well as ideological: Recent findings by the Global Disinformation Index (2019) showed that online ad spending on disinformation domains amounted to $235 million a year.

**Fig. 6.**
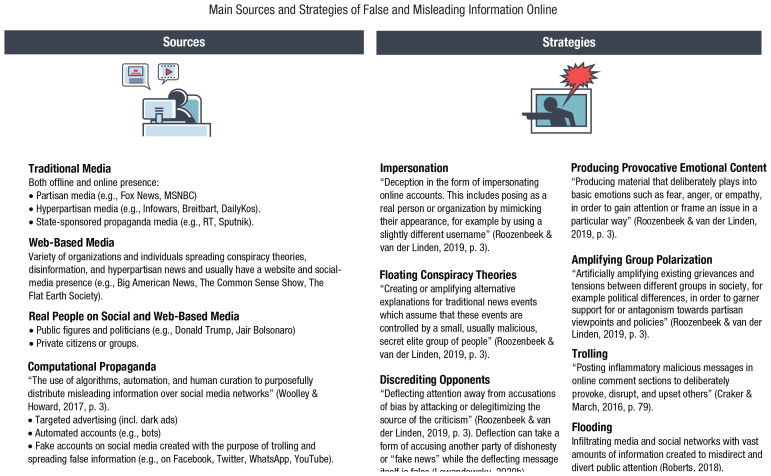
Main sources and strategies of false and misleading information in the digital world. Icons are used under license from Adobe Stock.

Creating and disseminating false information relies on several common practices that can be catalogued and used to develop tools to counteract disinformation (e.g., inoculation; see Roozenbeek & van der Linden, 2019, and the Inoculation: Boosting Cognitive Resilience to Misinformation and Manipulation section). Figure 5 lists the main categories of false and misleading information in the digital sphere; Figure 6 lists the main sources and strategies used for its creation and dissemination. We have compiled these classifications from a wide range of sources (indicated in the figures). One likely reason for controversies in the literature on the impact and significance of false information is the use of narrow definitions of fake news that exclude many manipulative sources as well as half-truths and other misleading techniques. At the same time, the “type of misinformation on the margins” (Warzel, 2020, para. 33)—that is, “believable information [that] is interspersed with unverifiable claims” (para. 37)—is the most difficult to trace, debunk, and verify. We have therefore chosen to include a variety of sources and types of false and misleading information instead of focusing on a narrow definition of fake news or a more abstract definition of “information disorders.”

Propaganda, rumors, conspiracy theories, and other kinds of misleading information are not novel phenomena, nor are they exclusive to online environments (see Uberti, 2016): As early as 1275, England’s First Statute of Westminster (English Parliament, 1275) outlawed spreading false news, stating that “none shall report slanderous news, whereby discord may arise” (Chapter 34). Numerous fake-news stories were published in newspapers in the 19th century, including the Great Moon hoax published in the New York tabloid *The Sun* in 1835. However, what distinguishes online propaganda and misinformation is the new medium itself. Besides having the capacity to spread misinformation further and faster, online environments offer new tools for computational propaganda that rely on the combination of algorithms and automation (e.g., bots) with human curation to flood social-media networks with misleading and polarizing content (Bradshaw & Howard, 2019; Woolley & Howard, 2017). The scope of false information and the speed with which it proliferates online is deeply connected to the nature and technical capabilities of online networks (Bounegru et al., 2018). Yet to fully understand the dissemination dynamics of false information, one must also consider how it hits specific hot buttons in people’s psychological make-up and who might be particularly susceptible to believing and sharing false news and rumors.

#### Psychology of fake news and receptivity to false information

Recent research has found that false news on Twitter spreads faster, deeper, and broader than does truth (Vosoughi et al., 2018).^17^ Fake news appears to press several psychological hot buttons. One is negative emotions and how people express them online. For instance, Vosoughi et al. (2018) found that false stories that “successfully” turned viral were likely to inspire fear, disgust, and surprise; true stories, in contrast, triggered anticipation, sadness, joy, and trust. The ability of false news to trigger negative emotions may give it an edge in the competition for human attention, and digital media may, as Crockett (2017) argued, promote the expression of negative emotions such as moral outrage “by inflating its triggering stimuli, reducing some of its costs and amplifying many of its personal benefits” (p. 769). More generally, people are more likely to share messages featuring moral–emotional language (Brady et al., 2017). It is possible that this type of loaded language and content, which feeds on humans’ negativity bias (i.e., the human proclivity to attend more to negative than to positive things; Soroka et al., 2019), succeeds in “attentional capture”—that is, it manages to shift cognitive resources to particular stimuli (Brady et al., 2019).^18^

Another hot button that fake news can press is the human attraction to novelty and surprise. Anything that is new, different, or unexpected is bound to catch a person’s eye. Indeed, neuroscientific studies suggest that stimulus novelty makes people more motivated to explore (e.g., Bunzeck & Düzel, 2006). Vosoughi et al. (2018) found that false stories were significantly more novel than true stories across various metrics. They also found that people noticed this novelty, as indicated by the fact that false stories inspired greater surprise (and greater disgust). One interpretation of these findings is that falsehood’s edge in the competition for limited attention is that it feeds on a highly adaptive human bias toward novelty.

Still another factor in the dissemination dynamics of false and misleading information is that the business models behind social media rely on immediate gratification; deliberation and critical thinking slow users down, which is generally detrimental to a social-media organization’s internal goals. Recently, Pennycook and Rand (2019b) showed that insufficient analytic reasoning—rather than politically motivated reasoning—is what appears to drive people’s belief in fake news.^19^ People might believe fake news because they are discouraged from taking the time to think critically about it—but their political leanings affect whether they share it: “Participants were only slightly more likely to consider sharing true headlines than false headlines, but much more likely to consider sharing politically concordant headlines than politically discordant headlines” (Pennycook et al., 2019, p. 3; see also Pennycook et al., 2020). Deliberation and analytic reasoning thus seem to play a role in judging accuracy, but other factors contribute to sharing information, including one’s political loyalty. Political partisanship has also been shown to be a significant factor in selective sharing of news (Weeks & Holbert, 2013) and fact-checking messages (Shin & Thorson, 2017). Moreover, ideologically motivated reasoning has been shown to play a role in endorsement of conspiracy theories (Miller, Saunders, & Farhart, 2016, p. 837); U.S. conservatives are significantly more likely than liberals to endorse specific conspiracy theories or to espouse conspiratorial worldviews in general (van der Linden et al., 2020).

A final factor in the dissemination dynamics of false information is which people are especially susceptible to it. Several recent studies have shown that increasing age is associated with increasing susceptibility to false information (see Table 3). A study of fake news on Facebook found that Americans over age 60 were much more likely to visit fake-news sites compared with younger people (Guess et al., 2019). Furthermore, the vast majority of both shares of and exposures to fake news on Facebook were attributable to relatively small fractions of the population, predominantly older adults (Grinberg et al., 2019). Allen et al. (2020) also found that false news was “more likely to be encountered on social media . . . and that older viewers were heavier consumers than younger ones” (p. 4). Brashier and Schacter (2020) argued “that cognitive declines alone cannot explain older adults’ engagement with fake news” (p. 321), but that gaps in digital literacy and social motives may play a bigger role. The role of analytic reasoning and deliberation may also offer hints as to who is particularly susceptible to false information. For instance, it is possible that users who rely more on reason than on emotions when making decisions may be less vulnerable to fake news; indeed, there is some evidence that is consistent with this possibility (Martel et al., 2020).

### Distracting environments

We now turn to a final challenge of online environments: the way they shape not only information search and decision-making but also people’s ability to concentrate and allocate their attention efficiently. As early as 1971, Herbert Simon understood that in an information-rich world, an abundance of information goes hand in hand with a scarcity of attention on the part of individuals and organizations: “A wealth of information creates a poverty of attention and a need to allocate that attention efficiently among the overabundance of information sources that might consume it” (Simon, 1971, pp. 40–41). Information overload and scarcity of attention became even more salient with the rapid evolution and proliferation of the Internet and media technologies. The original goals behind the Web were to create a user interface that would facilitate access to information and to simplify the process of information accumulation in the interconnected online space (Berners-Lee et al., 1992). Organizing information and making it accessible is also part of Google’s official mission statement (Google, 2020).

However, as new informational environments evolved and business models of Internet companies were refined, the goals and incentives of Internet design shifted as well. Human collective attention became a profitable market resource for which different actors compete. Fierce competition for human attention has led to the growing fragmentation of collective attention, with ever greater proliferation of novelty-driven content and shorter attention intervals allocated to particular topics (Lorenz-Spreen et al., 2019). By analyzing the dynamics of collective attention that is spent on cultural items such as Twitter hashtags, Google queries, or Reddit comments, Lorenz-Spreen et al. (2019) showed that across the past decade, the rate at which the popularity of items decreased or increased has grown. For example, in 2013, a hashtag on Twitter was popular on average for 17.5 hr; in 2016, its popularity lasted only 11.9 hr. The authors’ explanation is that when an excess of information meets limited attentional capacities, people’s thirst for novelty leads to accelerated ups and downs for each item and a higher frequency of alternating items. In other words, the amount of collective attention allocated to each single topic is decreasing and more topics are attended to in the same amount of time.

In the online world, the ability to concentrate becomes even more compromised when one’s surroundings are full of distracting stimuli that, by buzzing, ringing, or flashing, constantly call for attention. Moreover, digital environments are no longer constrained to desktop screens but are becoming increasingly integrated in people’s daily routines through a variety of smart devices. Unsurprisingly, these environments, which breed constant distraction and interruption, has led to “distracted minds” (Gazzaley & Rosen, 2016). Even the mere presence of a smartphone can occupy attentional resources and reduce cognitive ability (Ward et al., 2017), and smartphone notifications disrupt performance on attention-demanding tasks even when people are not actively attending to their phone, arguably because of mind wandering (Stothart, Mitchum, & Yehnert, 2015). In other words, when limited resources are shared between different sources competing for human attention (e.g., task-specific actions and task-irrelevant thoughts), ability to concentrate on the task suffers and performance drops.

Likewise, media multitasking—simultaneously attend-ing to several media sources, such as TV, text messages, and websites—is becoming more and more common among not only younger people but also older people (C. Rosen, 2008). Studies of high school and university students showed that the typical student could not stay focused on a task for more than 3 to 5 min without checking their messages or browsing the Web (L. D. Rosen, Carrier, & Cheever, 2013); in addition, multitasking is particularly pronounced when people read on a screen rather than in print (Carrier et al., 2015). A study by Ophir, Nass, and Wagner (2009) demonstrated that individuals who frequently multitask are more distracted by the multiple media they consume and have more difficulties in cognitive control over their attention; for instance, they show greater difficulty in filtering out irrelevant stimuli from the environment or from their memory. A review of studies on multitasking shows that switching attention between tasks instead of concentrating on one specific task not only increases the time spent on a task but also negatively affects performance (Uncapher & Wagner, 2018).^20^

In the zero-sum race for finite human attention, modern Internet technologies are designed to be appealing, addictive, and distracting (see Harris, 2016). Take, for instance, Facebook, which provides users with many types of rewards, including positive feedback in the form of “likes” and shares, social reinforcements in messages and comments, and friend requests. As Meshi et al. (2015) noted, “even minimalistic cues of social success such as these may activate our brain’s reward system, and keep us coming back to Facebook for more” (p. 774)—not unlike in Skinner’s operant-conditioning experiments with rats and pigeons (“virtual Skinner boxes”; Davidow, 2013), but this time with humans as the subjects.

Indeed, some (e.g., Harris, 2016; Wu, 2016) have suggested that Internet companies may be using behaviorist research on operant conditioning and schedules of reinforcement (e.g., Ferster & Skinner, 1957) to reward and maintain distracted online behavior (e.g., playing video games or checking updates on social media). Jonathan Badeen, cofounder of the online dating app Tinder, recently acknowledged that its algorithms were inspired by this behaviorist approach (Reynolds, 2019). Reinforcements in those cases are messages, likes, matches, comments, or any desirable content that is delivered at irregular intervals and that prompts users to constantly refresh their feeds and check their inboxes. Furthermore, there is initial suggestive evidence that people’s behaviors on social media are consistent with reward learning. Using four large social-media data sets,^21^ Lindström et al. (2019) demonstrated that reward learning theory can also model human behavior on social media, which, according to the authors, “exhibited a signature pattern of reward learning, such that computational models inspired by RL theory, originally developed to explain the behavior of non-human animals, could quantitatively account for online behavior” (p. 22). By focusing on the timing of social-media posts, their analyses showed that “people dynamically adjust their social media behavior in response to their own social rewards, as predicted by reward learning theory” (p. 17). Neuroscientific research also suggests that receiving positive feedback on social media (e.g., in the form of “likes”) is associated with activity in the brain’s reward network (Sherman et al., 2016), especially in regions associated with reward processing and prosocial behavior (Sherman et al., 2018).

**Fig. 7.**
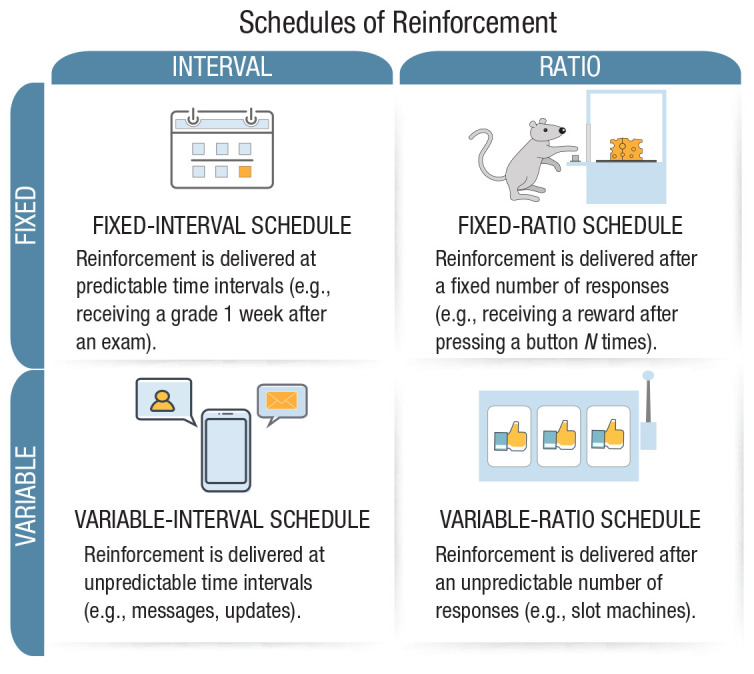
Four classes of schedules of reinforcement. The operant-conditioning chamber (also known as the Skinner box) was used to study animal behavior by teaching an animal (e.g., a rat) to perform certain actions (e.g., pressing a lever) in response to a controlling stimulus (e.g., a light signal) reinforced by a reward (e.g., food). Different schedules of reinforcement were studied to see which would create steady and high rates of response behavior. By analogy, “virtual Skinner boxes,” such as social media or online gaming offer their users rewards (e.g., likes or reaching another level in a game) at varying intervals to reinforce and maintain the desired behavior. Icons are used under license from Adobe Stock.

According to the operant-conditioning approach, the strength of behavior depends not only on the reinforcement, but also on the intervals or schedules at which rewards are delivered (Fig. 7). Although fixed schedules depend on rewards being delivered at predictable time intervals (fixed-interval schedules) or after a certain number of attempts (fixed-ratio schedules), in variable-interval schedules, reinforcements are delivered at time intervals that are unpredictable from a subjective perspective (e.g., checking text messages that arrive at unpredictable times). Variable-ratio schedules involve reinforcement after an average (but not fixed) number of responses (e.g., winning a prize after a variable number of attempts). Slot machines and lottery games are typical examples of variable-ratio schedules that maintain behavior efficiently (e.g., Thorens et al., 2012), as is online gaming (Ducheneaut et al., 2006). Variable-interval and variable-ratio schedules are both known to create a steady rate of responding; variable-ratio schedules produce the highest rates of response and variable-interval schedules produce moderate response rates (Domjan, 2018, p. 119). It seems that if rewards are difficult to predict, people tend to increase the rate of a particular behavior, perhaps hoping to eventually attain the desired reward.

To summarize, we distinguished four groups of challenges to human cognition and motivation in online environments. Our list of challenges is not exhaustive. Our focus here has been on urgent challenges to people’s agency, self-control, and autonomy of choice as well as to the civility and rationality of public discourse and ultimately the functioning of democratic societies. Many other issues raised by online environments and digital technology also deserve psychologists’ attention, such as the nature of the association between social-media use and individual well-being. The four challenges we reviewed are as follows:

- Human-made, ubiquitous, persuasive, and manipulative designs, which rely on dark patterns and hidden defaults, challenge the human capacity to exercise autonomous and informed choice. These practices affect not only how people access information but also—as is the case with privacy-intruding defaults—what information they agree to share.
- AI-assisted information architectures that filter information on the Internet and shape personalized information environments reduce agency and autonomy, amplify biases, and introduce obscurity into the automated decision-making processes.
- False and misleading information disseminated through social networks and digital media can have wide-ranging and serious consequences in the offline world. False information comes in many forms, including but not limited to fake news. In the race for people’s limited attention, false information systematically presses psychological hot buttons (e.g., emotional content and novelty) and exploits people’s lack of deliberation. Demographic factors, such as age, affect how receptive people may be to online falsehoods.
- Internet technologies are designed to be highly appealing, addictive, and distracting. Possibly armed with solid empirical and theoretical knowledge of operant conditioning, Internet companies compete in a zero-sum race for finite human attention. This has resulted in digital media that fosters distraction and attenuates people’s capacity for concentration and self-control.

## Behavioral Interventions Online: Nudging, Technocognition, and Boosting

**Fig. 8.**
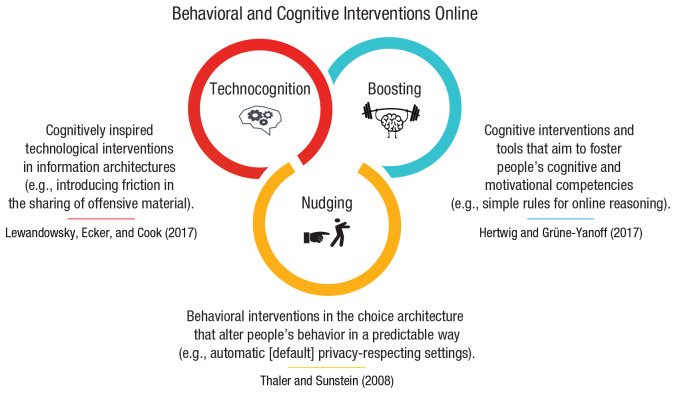
Types of behavioral and cognitive interventions for the digital world. The “nudging” icon is used under an Attribution 3.0 Unported (CC BY 3.0) license granted by Luis Prado at thenounproject.com. Other icons are used under license from Adobe Stock.

Although challenges loom large, they are not insurmountable. Insights and evidence from psychological science point the way to potential remedies. In this section, we summarize three types of evidence-based behavioral and cognitive interventions that can be applied to the digital world: nudges, technocognition, and boosts (Fig. 8).

### Nudging

Nudging is a popular approach to behavioral policy that harnesses the power of choice environments and the knowledge of human psychology to design choice architectures in ways that steer people’s decisions toward a greater individual or public good (Thaler & Sunstein, 2008). Nudging is based on the insight that it is possible to change people’s behavior—via their environment—without changing their minds. Nudging does not block, fence off, or significantly burden choices (as laws can do); rather, it proposes interventions that are meant to be easy, reversible, and cheap to implement. It thus represents a form of soft paternalism (also called libertarian paternalism). The target of these interventions is choice architectures (for a definition, see Table 1). In digital environments, the power of choice and information architectures over users’ behavior is even more significant than in the offline world. No online choice is ever made without predesigned context.

Nudging can be achieved in a number of ways—for example, by varying the order in which options are presented, thus changing their physical and cognitive accessibility. Rearranging food options in a cafeteria so that healthier foods are more accessible is meant to increase healthy food consumption (for systematic reviews, see Broers et al., 2017; Bucher et al., 2016). The preselected default option, a widely employed nudging technique, has a considerable impact on decisions (a meta-analysis by Jachimowicz et al., 2019, produced a medium-sized effect of *d* = 0.68). People are more likely to accept a preselected option than to select a different one (Jachimowicz et al., 2019); this is due to the mechanism of endorsement (defaults are seen as signaling what the choice architect wants the decision maker to do) or endowment (defaults are perceived to reflect the status quo). Benevolent choice architects can harness this tendency for causes serving the public good, such as increasing organ donation rates (Johnson & Goldstein, 2003; but see Arshad et al., 2019), or the good of the individual, such as saving more money for retirement through automatic enrollment (Thaler & Benartzi, 2004).

Commercial choice architects in online and offline environments, however, are privy to the same architectural principles. However, their design decisions are typically motivated by maximizing the benefits to the service provider rather than to the consumer. Commercial nudging can drive people to inadvertently subscribe to undesirable content or consent to privacy settings that are inconsistent with their stated best interests (see the Persuasive and Manipulative Choice Architectures section; see also Thaler, 2018, on “sludge”). The success and ethical permissibility of nudging thus largely depend on the goals of the choice architects (commercial or public good) and their alignment with the goals and values of individuals. Difficulties arise not only in determining people’s best interests or true preferences but also in maintaining a balance between what is best for different actors (individual decision makers, commercial bodies, political institutions) and society at large. Another critical issue in discussions on nudging is the assumed and actual role of human autonomy. Nudges do not eliminate available options and are easily reversible (Thaler & Sunstein, 2008, p. 236). Yet they substitute autonomous choice with preselected “rational” decisions to overcome people’s cognitive biases and inadequate decision-making competencies. As Rebonato (2012) argued, even though nominal autonomy might be preserved, effective autonomy may be reduced (but see also Sunstein, 2015). Another unintended consequence of nudging may be its potential impact on policy support; low-cost nudges might displace support for high-cost measures (e.g., the introduction of a green-energy default nudge risks diminishing support for a carbon tax; see Hagmann et al., 2019).

*Educative nudges* constitute a category of nudging that is explicitly respectful of human autonomy (Sunstein, 2015). As the name indicates, these interventions involve some form of education, for instance in the form of additional information (e.g., the nutritional quality of foods or the risks of smoking; Sunstein, 2016a, 2016b). In contrast to noneducative nudges, these interventions are transparent to people, engage their deliberate faculties, and preserve autonomy of choice—which may be why people prefer educative nudges. According to a nationally representative survey in the United States, a majority of people (between 55% and 74% across four topics; *n* = 430) consistently preferred educative versions of nudges (also referred to as System 2 nudges; e.g., statistical information about the risks of smoking, educational campaigns demonstrating advantages of green energy) over noneducative nudges (also referred to as System 1 nudges; e.g., graphic warnings on cigarette packaging, automatic enrollment in green-energy plans) when no information about their comparative effectiveness was presented (Sunstein, 2016b). Likewise, in another representative study in the United States (Jung & Mellers, 2016), participants viewed System 1 nudges such as defaults and sequential orderings less favorably than they viewed System 2 nudges such as educational opportunities and reminders.

### Technocognition

Technocognition is an approach proposed by Lewandowsky, Ecker, and Cook (2017) that offers a “cognitively-inspired design of information architectures” (Lewandowsky, Cook, & Ecker, 2017, p. 419). It suggests that a combination of insights from cognitive science and appropriate interventions in digital architectures can help in designing technological safeguards against the spread of false information or targeted adversarial manipulation. In digital environments, all choices are made in a predesigned context. Technocognition considers this design context through the lens of cognitive science. Cognitively inspired technological interventions can, for instance, introduce friction into the process of commenting on or sharing information. Consider the experiment launched by the Norwegian broadcaster NRK as a response to the problem of toxic commenting: Before readers could post a comment on an article, they had to pass a brief comprehension quiz on what they had read (Lewandowsky, 2020a; Lichterman, 2017). The friction created by increasing the entry cost for participating in online discussions was meant to foster deliberate thinking. Crucially, no one was censored in the process; once a person passed the quiz, they were free to comment as usual. Yet this measure, unlike a nudge, was meant to fence off certain behaviors unless the quiz was answered correctly—trolls were not expected to expend the effort required to pass the quiz.

Other examples of friction can be combined with prompts to engage analytical thinking. For example, Fazio (2020) showed that making people pause and think to explain why a certain headline was true or false can reduce their intention to share false headlines. This was not the case for true headlines. Pennycook et al. (2019, 2020) also found that introducing reminders about accuracy before sharing—thus subtly prompting people to attend to accuracy—can reduce people’s intention to share false headlines. In this case, technocognition and educative nudging converge.

A simpler version of friction can be used to prevent uncontrolled sharing cascades of false and misleading information. Instagram introduced an AI-powered feature in June 2019 that delays posts containing offensive comments by notifying users that their comment may be considered offensive and allowing them to cancel the post (Mosseri, 2019). Messaging app Telegram recently introduced a “slow mode” that enables group administrators to impose a wait period before users respond (Telegram Team, 2019). WhatsApp Messenger’s reportedly successful response to mob lynchings in India (see the False and Misleading Information section) was to limit the number of times a message can be shared to five chats—a feature that now applies to all users worldwide (WhatsApp, 2018). And most recently, Twitter introduced a new prompt (currently, as a test among users of Android operating systems) that encourages users who want to retweet an article that they have not opened yet to read it first (Twitter Support, 2020).

The underlying cognitive insights in these cases are twofold: First, limiting the number of chats to which a message can be forwarded or removing the share button from media posts introduced a delay, or cooling-off period. Cooling-off periods are known to affect people’s willingness to engage in an activity (for the effect of cooling-off periods on gun violence in the United States, see, e.g., Luca et al., 2017). Second, identifying a forwarded message as such provided a cue to users that the message originated not from a (potentially trusted) contact but from elsewhere. These interventions in the information architecture of social media, though small and easy to implement technologically, can have significant effects given the scale of these platforms—a promising point for designing appropriate technocognitive solutions in digital environments.

Let us stress, however, that similar techniques can also be used to restrict freedom of choice and communication on the Internet, as can be seen in the case of authoritarian regimes that use friction to limit citizens’ access to information (Roberts, 2018). It is therefore important to ensure that technocognitive interventions are designed with people’s best interests in mind and with public oversight.

### Boosting

Boosting is another class of cognitive interventions from psychological science. It responds to the challenge of rapidly changing digital environments by aiming to foster lasting and generalizable competencies in users (see also Hertwig, 2017; Hertwig & Grüne-Yanoff, 2017; Hertwig & Ryall, 2020). Boosts target individual cognitive and motivational competencies rather than immediate behavior (which is the target of nudges) and aim to empower people to make better decisions for themselves in accordance with their own goals and preferences. Boosting interventions can be directed at domain-specific competencies (e.g., understanding health information) and domain-general competencies (e.g., statistical literacy). They can target human cognition (e.g., decision strategies), the environment (e.g., information representation), or both (Hertwig & Grüne-Yanoff, 2017, p. 977). Moreover, in contrast to nudges, boosts specifically aim not only to preserve but also to foster and extend human agency and autonomy. Boosts are by necessity transparent because they require an individual’s active cooperation.

One example of boosting is a risk-literacy boost that can be applied to quickly educate people about relative versus absolute risks in, for instance, the health domain (Gigerenzer et al., 2007). Whereas benefits of drugs are often expressed in relative terms, such as “Drug X reduces the chance of stroke by 48%” (which suggests that the drug is highly effective), this information is incomplete and does not permit the user to judge the magnitude of the effect. Absolute risk information, by contrast, provides easy-to-understand information about the magnitude of the drug’s benefit: “Drug X reduces the chance of stroke from 28 per 1,000 to 15 per 1,000.” In this framing, the absolute reduction of stroke attributable to the drug is 13 per 1,000 people, or merely 1.3%. This risk-literacy boost is a simple, memorable rule—“always ask for health statistics to be translated into absolute numbers”—that can help people make more informed decisions about their health.

Boosting cognitive competencies online by redesigning the environment might involve changing the way information is presented to users or providing additional cues to existing information to improve the epistemic quality of online content (Lorenz-Spreen, Lewandowsky, et al., 2020). For example, such informational boosts can draw on research in algorithmic detection of false rumors; for example, Vosoughi et al. (2017) identified three categories of cues predictive of the veracity of online information (in this case, information shared on Twitter): people (who spreads the news), linguistic content (what words are used), and propagation dynamics (the shape of an information cascade). The most predictive cue in the study was propagation dynamics—a cue rarely detected by humans. This hidden cue, however, can be made transparent in design of visual aids (e.g., information icons on social-media posts) and can potentially improve the credibility of digital information (for more examples, see Lorenz-Spreen, Lewandowsky, et al., 2020).

Note that the additional information introduced in the environment is easily accessible but does not restrict users’ choices or activities. People can decide for themselves how much they want to engage with these information labels. In contrast to boosts that aim to foster long-term competencies, information labels are short-term interventions that provide quick and context-appropriate information. However, if one encounters them repeatedly, the development of long-term competencies could be spurred. At the same time, when evaluating interventions on the basis of informational cues (e.g., educational nudges and boosts that alter a decision maker’s environment), it is important to check their ecological validity and their actual capacity to make a difference. Some cues that are often cited as providing valuable information (e.g., URLs, publisher’s names) are in fact only weak signals that can easily be gamed in the current online ecosystem. For instance, highlighting the publisher of online articles (mainstream websites vs. misinformation websites) does not improve people’s ability to distinguish between accurate and inaccurate content (Dias et al., 2020). And, as Wineburg and Ziv (2019) argued, “dot-org symbolizes neither quality nor trustworthiness. It’s a marketing tool that relies on a widespread but false association with credibility.” Wineburg and McGrew (2019) suggested that it is more efficient to invest in teaching people how to verify information online (e.g., by boosting competence in lateral reading, to which we return later) than to rely on weak signals and cues. Indeed, interventions that trigger attention and deliberation should be more effective in the current attention-grabbing online ecosystem (Pennycook et al., 2019).

The entry costs for acquiring a boost should be as low as possible, so that many can engage with it. There is a role to be played by platforms and regulators to deliver easy-to-use cognitive tools to people. This also requires that people need to realize that there is a problem to begin with. But given survey results on trust in social media and the perceived prevalence of false information online (e.g., Newman et al., 2020), it is fair to say that many people have already woken up to at least some of the challenges discussed here. One might argue that the boosting approach may be limited in its need for active engagement and cognitive effort to develop or improve competencies. Indeed, unlike nudging, boosting does not bypass cognition and agency—it explicitly targets them. However, boosting is rooted in a different view of human psychology: It views people not as cognitive misers full of biases who are unable to make good decisions on their own but rather as admittedly bounded decision makers who have, however, the ability to learn and to rely on simple cognitive strategies that can adapt to uncertain environments (Hertwig et al., 2019).

Moreover, there is great heterogeneity in factors that matter for designing and choosing appropriate interventions for digital challenges, including individual demographic differences (e.g., age and education level) and other dimensions (e.g., affinity with technology, political attitudes, and people’s level of motivation to learn new competencies). Therefore, one should be careful to propose one-size-fits-all solutions. Indeed, some people might not be able or willing to engage with boosts, whereas others would be motivated to do so; some people might prefer policies that target deliberate processes over nondeliberative nudges (e.g., Jung & Mellers, 2016; Sunstein, 2016b). It is unlikely that a single solution that accommodates all users exists. For this reason, our vision is that of a toolbox of interventions that would reach different people and tackle different existing and emerging challenges.

It is also worth noting that the potential effects of any behavioral intervention—be it nudging, boosting, or technocognition—might be low. It is difficult to change people’s attitudes and behaviors, especially when there are so many intertwining factors in the real world and in human psychology that influence why people do what they do. Even the effects of what had been hoped to be a life-saving nudge—making organ donation the default option instead of requiring people to opt in (Johnson & Goldstein, 2003)—appear to fade away in the face of the realities of the world (e.g., objections of family members; see Arshad et al., 2019). Moreover, because digital environments are highly volatile, interventions in the choice architecture itself would inevitably be short-lived and subject to gaming. Under such adversarial conditions, betting on cognitive effort and empowered citizens might be less risky than relying on choice architectures that can be overridden at any moment by the uncertainty of the offline and online worlds. At the very least, boosting should complement regulations or nudges.

To summarize, it is possible to distinguish among a range of interventions, all informed by psychological science and behavioral sciences, that can be harnessed to respond to the four challenges of online environments outlined earlier. Conceptualizing and studying these interventions is a task of the highest order. As long as regulators fall behind the speed of change in digital environments and are hamstrung by the political power of Big Tech, interventions informed by scientific evidence will be crucial. We next turn to a map of boosting interventions in digital environments.

## Boosting Cognitive Competencies in Online Environments

**Fig. 9.**
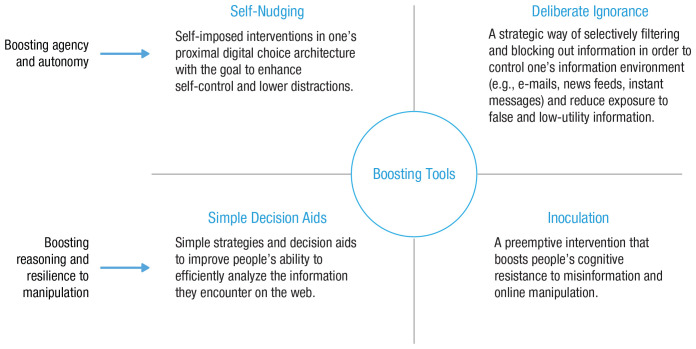
Map of boosting interventions for the digital world.

The interventions we review here are designed to simultaneously satisfy two constraints: (a) remedying specific problems in the digital world and (b) building on existing competencies or fostering new competencies. An important point is that different tools are adapted to counter specific challenges. For instance, social-media platforms exploit humans’ reward sensitivity to create hard-to-control habits that these platforms subsequently exploit. The best response to manipulative and persuasive choice architecture might therefore be to empower users to become choice architects of their own proximate digital environment (self-nudging) or self-restrict engagement with certain information sources (deliberate ignorance) rather than attempt to exercise a superhuman ability to detect and resist all attempts at influence. By contrast, false information and AI-powered persuasive techniques such as targeted political advertisement can best be met by people exercising existing competencies (e.g., reasoning and judgment of information reliability) or learning new ones (e.g., lateral reading). We thus identify two main groups of cognitive boosting tools (Fig. 9): (a) those aimed at enhancing people’s agency and autonomy in their digital environments (e.g., self-nudging and deliberate ignorance) and (b) those aimed at boosting reasoning and resilience to manipulation to accurately assess content encountered online (e.g., simple decision aids, inoculation). The effectiveness of some of these boosts has already been demonstrated experimentally; others are supported by evidence collected from neighboring areas of research in behavioral and cognitive sciences (e.g., research on nudging, self-control, and the use of simple heuristics in decision-making under uncertainty). These evidence-based and evidence-informed interventions can be presented to users, educators, and policy makers in the form of fact boxes, apps, and policy recommendations.

### Self-nudging: boosting control over one’s digital environment

The design of choice architectures that make online environments open to adversarial manipulation of user behavior can also be used by people to foster self-control and motivation. Online environments permit—although rarely encourage—a relatively high level of control over one’s choice architecture, such as setting one’s own defaults, adjusting notifications, installing ad blockers, and organizing one’s digital environment in a way that hinders interruptions and undesirable triggers. Users can take control over their digital surroundings and exercise freedom and agency by not being passive with regard to their environment. Accordingly, successful interventions in persuasive and attention-maximizing environments should aim to enhance people’s autonomy and their ability to control and shape their digital environments in ways that are consistent with their own goals. This does not mean that the responsibility for important features of the digital choice architecture would be shifted from companies or regulators to users. Taking at least some control of one’s proximate online environment must complement other policy measures (e.g., data privacy protection by default), not replace them—not least because it is effective only for those who are motivated enough to actively intervene in their own choice architecture.

One class of behavioral intervention that focuses on engaging with one’s proximate choice environment is self-nudging (Reijula & Hertwig, 2020). Self-nudging is a cognitive boost that fosters people’s competencies to design their proximate environment in a way that works best for them. Although nudging redesigns choice architectures to prompt a behavioral change, self-nudging empowers people to act as their own choice architects. For example, one can choose to implement a nudge in one’s own kitchen by moving tempting but undesirable foods to harder-to-reach places. In Duckworth et al.’s (2018) classification of self-control strategies, self-nudging falls into the category of self-deployed situational strategies. The approach of self-nudging draws inspiration from three sources. First and foremost, it has explicit roots in nudging and its emphasis on choice architecture—but, importantly, it aims to share the psychological knowledge built into nudges with the individual. Self-nudging can therefore benefit from the accumulated evidence on nudges such as defaults (e.g., Jachimowicz et al., 2019) or changes in cognitive and spatial accessibility (Thaler & Sunstein, 2008).

Another inspiration for self-nudging comes from economic research on commitment devices (Bryan et al., 2010; Rogers et al., 2014; Schelling, 1978), used predominantly to solve self-control problems. “Commitment devices attempt to enforce people’s voluntarily imposed restrictions until they have accomplished their goals, or their voluntarily imposed penalties for failing to accomplish their goals” (Rogers et al., 2014, p. 2065). In other words, a commitment device is a way to lock oneself into doing something that one might otherwise not be able to follow through with. One example is to define a health goal such as weight loss and to tell as many people as possible about when the goal must be reached and the penalty for not reaching it on time (e.g., donating to a political campaign one deeply dislikes).

Finally, self-nudging is also related to the notion of situational control (Duckworth et al., 2016), to research emphasizing the role of environment on habit formation (e.g., Wood, 2019), and to behavioral stimulus control—employed, for instance, in cognitive-behavioral therapy to treat insomnia or substance abuse (e.g., Edinger et al., 2001; for online addiction, see also Griffiths et al., 2014). Here, strategic changes are introduced in the environment to manage one’s exposure to stimuli that exercise control over one’s behavior. For instance, if a person is triggered by hyperpalatable stimuli (e.g., sugary food), removing them from the proximate environment or making them less accessible should strengthen the person’s ability to control urges. The same rationale can also be applied to one’s information diet. According to Wood (2019), the key to self-control in the digital domain is in taking control over the contextual cues that activate people’s use of technology (e.g., smartphones) and adding friction to make undesirable actions (e.g., excessive phone use) more difficult (pp. 234–235). In what follows, we briefly review three types of self-nudges that can be enlisted by people to nudge themselves away from distracting sources or make their desired options more easily available.

#### Self-nudging by adapting cognitive accessibility

**Fig. 10.**
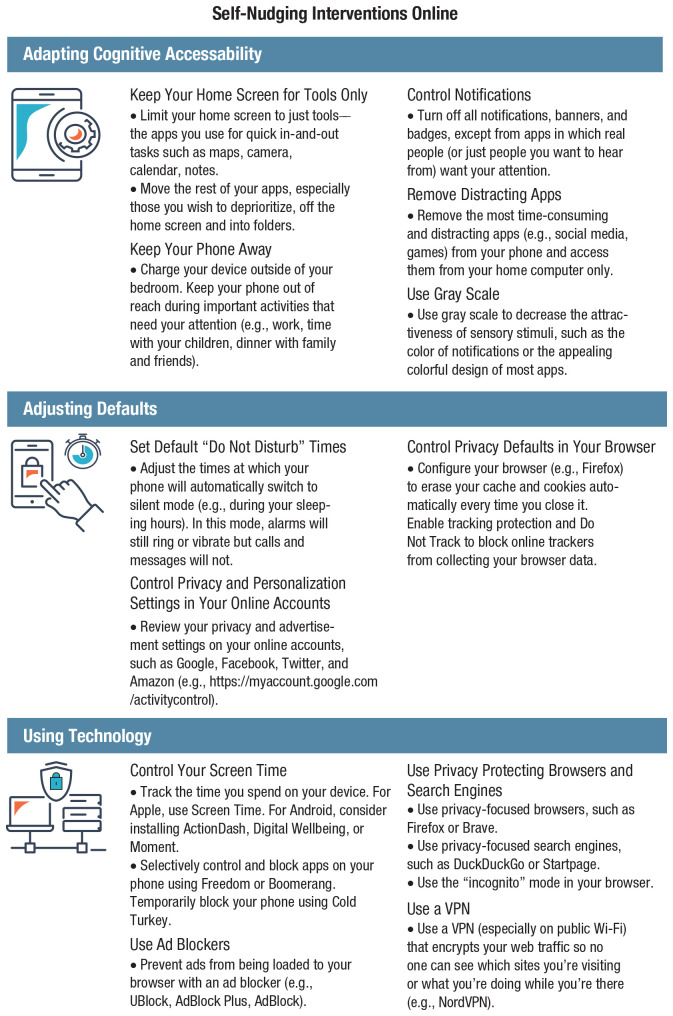
Self-nudging interventions in online environments. A summary of potential self-nudging interventions to enhance people’s control over their digital environments and their privacy protection online. Based in part on Center for Humane Technology (2019) and Epstein (2017). Icons are used under license from Adobe Stock.

The Center for Humane Technology (2019) suggests several steps that people can take to exercise more control over the time they spend on their devices. For example, the variable reinforcement schedules of notifications (see Fig. 7) can turn checking one’s phone into a powerful habit. People can control these distracting stimuli by turning off notifications for anything not coming directly from other people (e.g., news apps) or even by allowing notifications only from apps used by their most important contacts (e.g., enabling notifications for messenger apps they use with friends and family but disabling e-mail notifications). They could set specific times in which messages can be received, thereby reserving periods of time for concentrated work (see also Newport, 2016). This measure can also help convert variable schedules of receiving messages to fixed-interval schedules (which are known to elicit the lowest rates of responding), thereby potentially reducing messages’ addictive character. Further advice includes deliberately separating applications that, by one’s own standards, improve the quality of time spent online (e.g., educational podcasts) and those that do not. This can be achieved by rearranging one’s smartphone home screen so that only useful apps (e.g., podcasts and meditation apps, as well as tools such as calendars and maps) are displayed on the front page, whereas others (e.g., social media, games) are tucked away in folders (see Fig. 10, regarding adaptive cognitive accessibility). Other self-imposed interventions in one’s digital choice architecture include removing social-media apps from one’s mobile devices and accessing them from a home computer only or deliberately placing devices out of sight to reduce the cognitive accessibility of the most distracting platforms.

#### Self-nudging by adjusting defaults

Defaults, one of the most widely employed tools in the choice-architecture toolbox, are fertile ground for self-nudging. People can take control of their digital default settings, including privacy settings on social media and settings for personalized advertisements (e.g., https://myactivity.google.com). Although it might initially require some time, effort, and possibly even guidance to understand how default settings work and their considerable effect on people, self-command over defaults may prove to be a powerful way to return agency and autonomy to users (see Fig. 10, regarding adjusting defaults).

#### Self-nudging with the help of technology

One can also make use of apps (e.g., Digital Wellbeing, Cold Turkey, Freedom, and Boomerang; see Fig. 10, regarding using technology) that allow users to control how much time they spend on their phones, to schedule e-mails, or to block all notifications for a period of time to maintain focus. Being in control, the self-nudger decides which goals and tools to prioritize and which to move to the background. Another helpful tool in one’s choice architecture is a monitor of one’s habits of information consumption. Users who aim for balanced reading may install a browser that monitors the extent to which their reading history is consistent with their goal. The feedback from the browser widget would thus work as a self-deployed reminder of one’s epistemic goal (Munson et al., 2013).

Self-nudging is particularly suited to situations in which exercising self-control or resisting temptation is difficult or when a choice environment is toxic (i.e., when choice architects design highly addictive environments with nonbenevolent goals in mind; see Hertwig, 2017). Moreover, self-nudging enhances autonomy because it aims to put people in charge of determining their own goals and choice environments, thus bypassing the paternalism that can accompany the kind of nudging that capitalizes on people’s deficiencies rather than attempting to educate them. “Self-nudging means that people intentionally nudge themselves in order to self-regulate their behavior and break self-destructive habits. When the nudger and the nudged are . . . the same person, as in the case of self-nudging, autonomy and agency remain intact” (Hertwig, 2017, p. 155). A sense of agency is crucial for self-nudging. As a Pew Research Center survey showed, users who think they have more control over their news feeds tend to influence the content of their Facebook feeds more than users who think they have no control do (Smith, 2018a).^22^

Let us also highlight the potential limitations of self-nudging. Although a perceived sense of control is crucial for exercising agency, it should be backed up by appropriate affordances in the environment. As discussed previously, persuasive design can create an illusion of control and still nudge users away from privacy-friendly choices (e.g., Norwegian Consumer Council, 2018). Moreover, if individuals’ perceived control over the release of and access to private information is increased, it can, paradoxically, increase their willingness to disclose sensitive information (“the control paradox”; Brandimarte et al., 2013). For example, users can have a strong feeling of control on Facebook because they can change their default privacy settings and adjust who will see what type of information in their profiles. At the same time, they have very little control over the way in which the information they share will be used by the platform, by third-party applications, or even by their friends. We hold that self-nudging efforts should be complemented by reasonable regulations and online tools^23^ that not only give users more control over their digital environments (e.g., empowering them to customize the design of their own social-media news feed; see Lorenz-Spreen, Lewandowsky, et al., 2020) but also ensure that personal information is protected regardless of users’ actions.

Finally, self-nudging will hinge on the ability and willingness of psychologists and public choice architects to let citizens in on the secrets of nudging. The psychological principles behind nudges are not well known to the general public, even though they are relatively easy to explain and easy to understand: For instance, reduced cognitive accessibility is what keeps people from snacking on hard-to-reach cookies or opening the social-media apps they removed from their home screen. One possible—and certainly welcome—side effect of self-nudging may be that people become more aware of the extent to which mundane environmental factors, both online and offline, sway human behavior and become more curious about how they can turn their proximate environments into allies.

### Deliberate ignorance as information management device

In 1807, Thomas Jefferson condemned the “polluted vehicle” of newspapers, claiming that “the man who never looks into a newspaper is better informed than he who reads them; inasmuch as he who knows nothing is nearer to truth than he whose mind is filled with falsehoods and errors” (1999, p. 275). The current challenge of information overload and environments designed to compete for human attention by offering rewards and hyperpalatable stimuli brings new significance to this statement. The challenge is amplified by the proliferation of politically motivated agents that specialize in the cultural production of ignorance, including the organized campaigns that undermine the public’s perception of the scientific consensus around climate change to thwart policy initiatives (Oreskes & Conway, 2011; Proctor & Schiebinger, 2008). Technological advances have fostered novel methods for producing ignorance and putting the very existence of objective truths into question. The flooding technique is one such method. The Chinese government is estimated to create and post about 448 million social-media comments per year—not to address controversial issues or even to argue with critics of the party and the government, but rather to divert attention from real issues (e.g., the government’s lackluster response to natural disasters) toward trivial and scandalous stories that are injected online for the sole purpose of distracting the public from discovering government weaknesses (King, Pan, & Roberts, 2017; Roberts, 2018).

Readers of digital media face a constant trade-off between staying informed about current events and being exposed to an information environment in which numerous players (e.g., companies, advertisers, media, and policy makers) design hyperpalatable mental stimuli to hijack people’s limited attention. Much as obesogenic environments are replete with foods designed to offer maximal sensory pleasure, informationally fattening environments degrade consumers’ control and autonomy over the information they consume (Crawford, 2015). When low-quality clickbait stories, conspiracy theories, and fake news masquerade as meaningful information, epistemic abstinence becomes more rational than epistemic indulgence. In other words, more information is not always better. To manage information overload, one must ignore a large amount of incoming material and separate useful information from noise, false news, or harmful advice. In this context, deliberate ignorance can be used as one tool for information management (Hertwig & Engel, 2016, 2020).

Hertwig and Engel (2016) defined deliberate ignorance as the “conscious individual or collective choice not to seek or use information (or knowledge)”—particularly when the “marginal acquisition costs are negligible and the potential benefits potentially large” (p. 360). The idea that deliberate ignorance can be an ecologically rational strategy does not align with classical ideals of epistemic virtue and rationality (see Kozyreva & Hertwig, 2019), which presume that information and knowledge have intrinsic value for decision makers because they allow them to accumulate more evidence (e.g., Carnap, 1947), acquire better understanding, and ultimately make more informed and rational choices (e.g., Blackwell, 1953; Good, 1967). However, deliberate ignorance is a reasonable strategy in many situations—for instance, in the interest of impartiality and to shield oneself from biases (e.g., see MacCoun, 2020). One concrete example is the practice of blind auditioning for orchestras, in which candidates play behind a screen to hide their identities. As suggested by Goldin and Rouse’s (2000) analysis, the introduction of this policy has contributed to a substantial increase in the proportion of women in orchestras during the second half of the 20th century. Another example of deliberate ignorance: a person who has been diagnosed with a serious illness decides to not ask about a prognosis. This can be seen as an irrational avoidance of information fueled by the prospect of a bleak future. Alternatively, however, it can be viewed as the person’s affirmation of their informational autonomy and their legitimate desire to protect themselves from the weight of a menacing—and not necessarily accurate—timeline.

People deliberately ignore information for various reasons—for instance, to avoid emotional costs (e.g., choosing not to test for a rare genetic disease), to benefit from strategic ignorance (e.g., in negotiations), or to maximize suspense and surprise (see Hertwig & Engel, 2016). Hertwig and Engel (2016) also suggested that deliberate ignorance could be a mental device to boost cognitive sustainability and information management, especially in the digital world. They argued,

For humans, who are hardwired to monitor their environment, the ability to allocate one’s limited attentional resources reasonably is therefore becoming increasingly valuable in today’s world. Indeed, the ability to select a few valuable pieces of information and deliberately ignore others may become a core cultural competency to be taught in school like reading and writing. (p. 364)^24^

Online health information is an environment in which deliberate ignorance can be a helpful and reasonable tool for managing information. Although online access to health information can increase people’s knowledge and foster beneficial health-related decision-making (Jacobs, Amuta, & Jeon, 2017), the abundance of low-quality sources with user-contributed content—such as blogs, online forums, celebrity web pages, and social networking sites—puts people at risk of becoming victims of bad health advice or even conspiracy theories.^25^

Tempting, highly unrepresentative, and possibly even misleading information environments (e.g., health claims about miracle cures or breakthroughs that are too good to be true, inflammatory false claims that a vaccine causes mental retardation; see Thomas, Tandoc, & Hinnant, 2017) are difficult to navigate. One ecologically rational strategy in such environments is to abstain from seeking out these narratives; to avoid searching for one’s symptoms in search engines; and to ignore health advice from influencers, celebrities, or commenters in online forums. As in the case of blind auditioning, people may choose to shield themselves from eye-catching and tempting information that they can expect will distort their judgments. Given the scarcity of high-quality health information online, the ability to intentionally ignore low-value persuasive sources is an important skill. For example, Oxman and Paulsen (2019) identified only three websites that met their inclusion criteria for evidence-based aggregation of health information: Cochrane Evidence, Informed Health, and PubMed Health. Others are likely to exist (e.g., the National Health Service website in the UK). The next step would be to make the information on these websites publicly accessible and easily understandable (e.g., in fact boxes; see McDowell et al., 2016).

Let us emphasize two further key points: First, by advocating deliberate ignorance as a tool for the online world, we are not advocating for the proliferation of ignorance, echo chambers, and a return to the Dark Ages. An informed public remains the cornerstone of democracy, and widespread education is one of its highest achievements. Moreover, the accessibility of information offered by the Internet should be regarded as a public good. Our emphasis on deliberate ignorance as a tool for information management focuses on its strategic use to shield oneself from the excesses, traps, and information disorders of the current digital environment. Second, notwithstanding the common—and frequently justified—connotation of ignorance as an expression of mental indiscipline and indolence, deliberate ignorance (as conceptualized by Hertwig & Engel, 2020) requires cognitive and motivational resources (e.g., executive control, self-control). Online, informed deliberate ignorance also requires, somewhat ironically, knowledge—such as an understanding of what constitutes a reliable indicator of trustworthiness. It is therefore encouraging that laypeople—on average and across the political spectrum—have been shown to be collectively quite good at distinguishing between sources of lower and higher quality and to place more trust in media outlets with stronger editorial norms than in sources that are hyperpartisan or peddle fake news (Pennycook & Rand, 2019a). In the next section, we address strategies for discerning whether sources (e.g., websites) offer reliable information or not.

### Simple decision aids: boosting digital-information literacy

The concept of digital-information literacy encompasses the skills and competencies that are needed to successfully navigate the digital-information ecosystem so as to obtain, understand, and use information in a variety of online contexts (Sparks, Katz, & Beile, 2016). One aspect of digital-information and digital-media literacy is the ability to analyze and evaluate the information people encounter online, including judgment of information reliability or evaluation of sources and evidence. Kahne and Bowyer (2017) demonstrated a positive impact of media-literacy education on young people’s ability to evaluate news accuracy and to distinguish between evidence-based and false claims in online posts. Formal education in such skills is becoming increasingly more important, but it is also slow and effortful and is unlikely to engage older people. The idea behind simple decision aids for digital-information literacy is therefore to complement educational programs by providing people (young and old alike) with simple strategies and decision aids that can help evaluate information encountered online. The goal is to foster good habits that are as simple and automatic as washing one’s hands or scanning the crosswalk before making a turn (Caulfield, 2018).

One way to design such simple tools makes use of the skill set of professional fact-checkers, who are experts in evaluating the reliability of information. In order to develop a set of rules based on this skill set, researchers from the Stanford History Education group (Wineburg & McGrew, 2019) asked 45 participants (10 professional fact-checkers, 10 history professors, and 25 undergraduate students) to evaluate the trustworthiness of information online. Wineburg and McGrew (2019) argued that the key to experts’ success in fact-checking is their strategy of lateral reading, a heuristic rule that allows them to “read less and learn more” by looking to verify the claim outside of the original post. Contrary to the professors and students, who focused on the information source itself, fact-checkers (the most successful group of participants across several fact-checking tasks) spent most of their time verifying the source and the evidence behind the claim by checking information about it on the Web. “Instead of spending precious minutes scouring an unfamiliar site, checkers left it immediately. They ‘read laterally,’ opening up multiple tabs across their screens and researching the organization. They learned most about a site, paradoxically, by leaving it” (Ziv & Wineburg, 2020). In a similar vein, Graves (2017) attested that the key to professional fact-checkers’ analysis lies “in discovering a claim’s origin and reconstructing its spread” (p. 525).

**Fig. 11.**
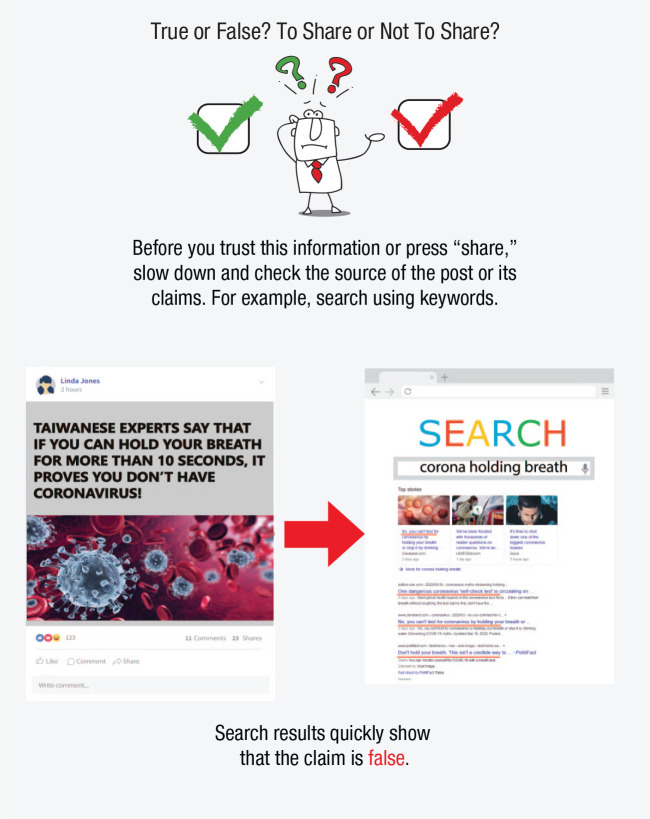
A simple lateral-reading boost. Based on research by the Stanford History Education Group (Breakstone et al., 2018; McGrew et al., 2019; Wineburg & McGrew, 2019). Icons are used under license from Adobe Stock.

Drawing inspiration from fact-checkers’ strategies, researchers identified simple rules geared at boosting competence in civic online reasoning. This competency includes three subcompetencies: evaluation of the source, evaluation of the evidence, and lateral reading. One way of representing these competencies is through simple questions: (a) Who is behind this information? (b) What is the evidence? (c) What do other sources say? (Breakstone et al., 2018, p. 221). McGrew et al. (2019) found that after two 75-min lessons on evaluating the credibility of online sources (an extended version of the three questions outlined above), students in the treatment condition (*n* = 29) tested on their online reasoning skills were more than twice as likely to score higher at posttest than at pretest, whereas students in the control condition (*n* = 38) were equally likely to score higher or lower at posttest than at pretest, indicating that the intervention was successful. As a quick boosting intervention, rules based on these questions can be presented in the form of simple tips on how to verify claims in, for instance, users’ social-media feeds (see Fig. 11 for an example).

Another set of digital literacy rules has recently been introduced to improve people’s ability to distinguish between false and mainstream news. This intervention—Facebook’s “Tips to spot false news”—aims to provide simple rules that help people identify suspicious information and false news. The tips include, for example, advice to be skeptical of catchy headlines, to investigate the “about” page and the URL, and to look for other reports on the news claim (Facebook, n.d.). The results of a randomized controlled study showed that exposure to this intervention reduced the perceived accuracy of both mainstream and false news headlines in the United States and India (with the exception of rural populations), but effects on false news were significantly larger although still relatively modest (in the United States, the perceived accuracy of false headlines decreased by nearly 0.3 points on a 4-point scale). Note that the improvement in performance in headline-accuracy rating did not depend on the headlines’ alignment with respondents’ political predispositions (Guess, Lerner, et al., 2020).

Further examples of simple decision aids that can be designed to foster better information literacy are fast-and-frugal decision trees (FFTs; Luan et al., 2011; Martignon et al., 2008). Already in use in a variety of domains, including medicine, finance, law, and management, FFTs provide comprehensive prescriptive guides for real-world decision-making (Hafenbrädl et al., 2016). They rank decision criteria in the order of importance and offer a potential exit at each point. To make a decision, a person goes through the cues sequentially. For example, medical professionals can use a simple decision tree to quickly categorize trauma patients into those who need immediate medical attention and those whose treatment can be delayed (one such system, called simple triage and rapid treatment [START], was used in New York City hospitals during the World Trade Center attack in 2001; see L. Cook, 2001). Cues in this case are framed as questions: Is the patient walking? If yes, delay treatment; if no, proceed to the following cue. Implementing and understanding an FFT is easy; it requires nothing more than knowledge of the order of the cues and their exit conditions.^26^

**Fig. 12.**
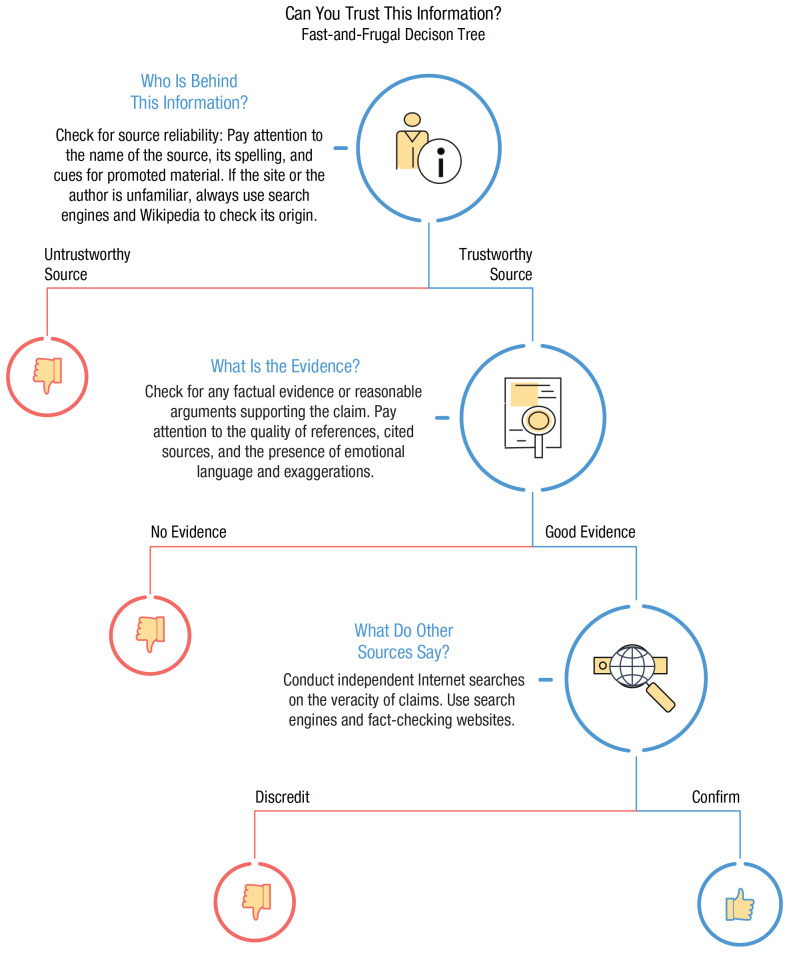
“Can you trust this information?”: This fast-and-frugal decision tree provides users with three crucial steps for evaluating the trustworthiness of information online. Based on research by the Stanford History Education Group (Breakstone et al., 2018; Wineburg & McGrew, 2019). Icons are used under license from Adobe Stock.

There are not many examples of simple decision aids for the online domain. But one short intervention has already been applied to improve people’s ability to use linguistic cues to distinguish between authentic and fictitious online reviews (Banerjee et al., 2017). Likewise, FFTs could be designed and tested as decision aids to choices such as whether to trust information encountered online. Figure 12 shows a potential decision tree based on the rules for fact-checking identified by Breakstone et al. (2018) and Wineburg and McGrew (2019). The FFT advances through the cues sequentially and ends when the answer is “no,” which indicates that the information is not trustworthy and should not be shared. It is noteworthy that a decision can often be made at the first step (which contributes to the FFT’s frugality) because it usually involves the best cue. FFTs work best with strong cues or signals, but in some cases a combination of weak signals—such as the top-level domain (e.g., .com or .gov), how the social-media name is spelled, the “about” page, and cues for verified accounts or promoted material—can be used (with the help of the tallying strategy). However, all these signals must be taken with caution. For example, a fishy top-level domain (e.g., com.co) is a signal that the source may be untrustworthy, but the opposite is not necessarily true (e.g., a .gov domain does not guarantee trustworthiness). It is clear that cues for trustworthiness can be gamed, and fake news websites can appear to be as genuine and well designed as the websites of real news organizations. That is why strong negative signals such as an unfamiliar website should be taken seriously, and unfamiliar sources should always be verified using the lateral-reading strategy. As a general rule of thumb for constructing FFTs, cues that are difficult to game should take precedence over those that are easy to game.

Like any cognitive tool in the toolbox of digital decision makers, simple decision aids must be used under appropriate conditions. For example, lateral reading is an effective tool for verifying the information encountered on a suspicious website or social-media feed, but it may not be the best strategy for reading trusted material that benefits from concentration and focus on one source. Likewise, decision trees are appropriate tools for dichotomous decisions (e.g., whether to trust or share a news item or not) but they might not be helpful for complex choices that require more sophisticated deliberation.

### Inoculation: boosting cognitive resilience to misinformation and manipulation

Another cognitive intervention against false information and online manipulation is *inoculation*, also known as *prebunking*. It targets people’s ability to recognize misleading or manipulative strategies before they encounter them face-to-face or online. Metaphorically speaking, if disinformation is a disorder, then inoculation can immunize people against certain strains of false and misleading information. Inoculation is preemptive: It aims to expose people to misleading or manipulative strategies and to neutralize their disruptive potential before people actually encounter them in the world (for more on the inoculation theory, see Compton, 2013; McGuire & Papageorgis, 1961). Inoculation differs from debunking strategies, which refute false claims only after they have been seen or heard; it is thus especially valuable, given that disinformation is often resistant to debunking after the fact (J. Cook et al., 2017; Lewandowsky et al., 2012). Furthermore, unlike topic-specific debunking, inoculation is intended to instill domain-general competence in recipients to enable them to see through attempts at manipulation (Roozenbeek & van der Linden, 2019), making it a particularly suitable cognitive strategy when fact-checking or evidence-based refutation is costly or unavailable.

According to J. Cook et al. (2017) there are two components to inoculation: first, an explicit warning about a potential threat of disinformation or manipulation—for example, a warning about attempts to cast doubt on the scientific consensus on climate change that create a chimerical set of “experts” who disagree with the consensus. The second step refutes an anticipated argument, thus exposing the disinformation strategy and rendering its deceptive nature transparent. In our climate-change example, this could take the shape of an illustration and an explanation of a particular deceptive technique used to question a scientific consensus or otherwise manipulate the public (J. Cook et al., 2017, p. 4). In the study by J. Cook et al. (2017), the inoculation consisted of showing participants the “fake experts” strategy used by the tobacco industry in the 1960s (a tobacco ad with the text “20,679 Physicians say ‘Luckies are less irritating’”). The same strategy was used by climate-science denialists: The Oregon Petition denied human-caused effects on the Earth’s atmosphere and was signed by 31,000 alleged experts, of whom 99% had no expertise in climate science. By exposing participants to a weakened version^27^ of disinformation, this intervention provided them with a counterargument. The efficacy of inoculation in preventing acceptance of disinformation has been established in several experiments (J. Cook et al., 2017; van der linden, Leiserowitz, et al., 2017) and inspired the creation of Bad News, an educational game on fake news (Roozenbeek & van der Linden, 2018, 2019).

The Bad News study aimed to extend the effects of inoculation beyond a particular topic (such as climate change) and develop a “broad-spectrum vaccine” against disinformation (Roozenbeek & van der Linden, 2019, p. 2). It focused on the tactics commonly used to produce disinformation, rather than on the content of a specific disinformation campaign. The study provided an active type of inoculation (see Table 4) by having participants play a game (https://getbadnews.com/) in which they learned six strategies often used to spread disinformation (according to NATO Strategic Communications Centre of Excellence, 2017): impersonating people or famous sources online, producing provocative emotional content, amplifying group polarization, floating conspiracy theories, discrediting opponents, and trolling (summarized in Fig. 6). The underlying idea of the game is that people train to become expert manipulators by applying different disinformation techniques. In doing so, they develop competence in detecting manipulation, which will help them realize when manipulative strategies are being applied to them in the future. The game environment represents a weakened form of real-world social media (where people are apt to encounter false information). The inoculation effects of the Bad News game were observed by comparing preintervention and postintervention credibility ratings of various fake-news items (*n* = 14,266; *d* = 0.52 average across all items). The effects were most pronounced for individuals who had been more susceptible to fake-news headlines in the first place (*d* = 0.89). Similar effect sizes (*d* = 0.60) were found in a follow-up randomized controlled study by Basol et al., (2020) that compared the efficacy of the Bad News game intervention with that of a control condition. Both studies (Basol et al., 2020; Roozenbeek & van der Linden, 2019) found that none of the observed main effects “revealed an interaction with political ideology, suggesting that the intervention works as a ‘broad-spectrum’ vaccine across the political spectrum” (Basol et al., 2020, p. 5).

**Table 4.** Structure of Experimental Inoculation Interventions

Inoculation Type 1 (passive)	Warning about potential misinformation or manipulation (e.g., about attempts to cast doubts on scientific consensus on climate change)	Refutation of an anticipated argument in a weakened form (e.g., an example and an explanation of the “fake experts” strategy)	Postintervention exposure (e.g., expose participants to the same strategy used by climate science denialists)
Inoculation Type 2 (active)	Preintervention test (e.g., ratings of fake news credibility)	Active learning (e.g., the Bad News game, which aims to present main disinformation strategies in a weakened, fun way)	Postintervention test (e.g., credibility ratings of fake news)

Inoculation aims to boost cognitive resilience to disinformation and manipulation (van der Linden, Maibach, et al., 2017). As is the case with all the interventions we have discussed, it is an efficient strategy when it fits particular challenges in the environment and the cognitive competencies involved. Inoculation interventions must be based on an understanding of the manipulative strategies being used online and how they work. Furthermore, people must be willing to be inoculated—that is, to take the time to learn about these techniques. Another limitation of inoculation is that it is ineffective in the face of unexpected or novel deceptive techniques. Thus, as with vaccines in the physical world, it makes sense to be prepared for the most insidious and common methods of online manipulation and to regularly update inoculation techniques. The logic of inoculation can be extended beyond misinformation to other challenges—for instance, helping people detect manipulation through personalized political advertisement that exploits people’s psychological identities and vulnerabilities (Lorenz-Spreen, Geers, et al., 2020).

## Conclusion: From Psychological Science to the Internet for Citizens

Technological innovations have frequently been associated with dystopian fears. As far back as 370 BCE, thinkers such as Socrates were deeply concerned about the detrimental consequences of writing:

In fact, it [writing] will introduce forgetfulness into the soul of those who learn it: they will not practice using their memory because they will put their trust in writing, which is external and depends on signs that belong to others, instead of trying to remember from the inside, completely on their own. . . . Your invention will enable them to hear many things without being properly taught, and they will imagine that they have come to know much while for the most part they will know nothing. (Plato, ca. 370 B.C.E./1997, pp. 551–552)

Today’s concerns about, for instance, the potential effects of Google on memory (e.g., Sparrow, Liu, & Wegner, 2011) and comprehension, or about digital amnesia or digital dementia (e.g., Spitzer, 2012), echo Socrates’s fear of forgetfulness and shallow comprehension. Socrates was not necessarily wrong—it might well be the case that the capacity of human memory has fundamentally changed from the time knowledge was transmitted orally. Yet he did not foresee the wide range of benefits—including the invention of the Internet—that were rendered possible by this new form of com-munication.

Honoring this lesson, we are cognizant of the risk of conjuring up dystopian fears. The current and future benefits of the digital revolution are immense. Yet hopes for a digital utopia did not survive the harsh light of reality, and the original optimistic narrative of liberation technology (Diamond, 2010) has been gradually replaced by one that is raising grave concerns about “surveillance capitalism” (Zuboff, 2019). A growing body of evidence reveals worrying implications of the digital transformation, and at least four aspects of the transformation cause particular concern.

First, unlike previous communication innovations, which permeated societies on time scales of centuries (e.g., writing) or decades (e.g., telephony), today’s digital transformations occur at a breathtaking pace. Apps can appear outdated within a few months, and the life cycle of information technologies is notoriously short. The comparatively slow pace of academic research, with its cycle of prolonged peer review and revision, cannot fully capture, let alone influence, those trans-formations.

Second, the problem of speed is compounded by the degree of mutation that technology can undergo. Whereas the psychological affordances of writing changed little during the transition from parchment and quill to paper and pencil, new digital technologies can create new psychological affordances in an instant. For example, the seemingly trivial addition of a “retweet” button has made it possible for a small number of people—or indeed, nonhuman “bots”—to trigger global informational cascades (e.g., Bastos & Mercea, 2019).

Third, the implications of those mutations cannot be anticipated. WhatsApp did not anticipate that the ease with which material can be shared would contribute to mob killings, and Facebook probably did not anticipate that a platform designed for staying in touch with friends and family could end up influencing the outcome of elections through dark ads and misinformation (Jamieson, 2018).

Finally, and perhaps most troubling, is that this digital transformation is occurring in what is largely a regulatory vacuum. There is nothing to stop platforms from radically altering their interfaces overnight, with unknown consequences for society and democracy—a situation recently brought into focus by Facebook’s decision to allow the distribution of false statements in political advertisement under the argument of free speech protection (Facebook, 2019).

A recent report from the RAND Corporation (Mazarr et al., 2019) condensed those concerns into a number of future scenarios, described under the umbrella term of the “emerging risk of virtual societal warfare.” Mazarr et al. (2019) pointed to associated social trends, such as declining faith in institutions that help to sustain social truths that are generally agreed upon (e.g., the media), weakened measures of social capital (e.g., social trust and civic engagement), increased partisan polarization across many countries, a rise in populist movements and, last but not least, what various scholars (e.g., Specter, 2009, p. 33) have described as a sense of alienation and a loss of agency and ontological security (Giddens, 1991). People’s trust in social institutions, their interpersonal exchanges, the stability and reliability of facts, and even their sense of shared reality are being undermined. One of the future digital scenarios considered by Mazarr et al. is called “the death of reality.” Envisaged for 2023, it is the point at which the “ability to manufacture seemingly tangible reality from scratch has . . . become commonplace” (p. 99). Present-day antecedents for this scenario can be found in the radical constructivist ontology of truth employed by practitioners of “post-truth” discourse (Lewandowsky, 2020b). Arguably, this scenario can materialize only within a digital information architecture that permits people to personalize all of reality along with their preferences for deodorants.

**Fig. 13.**
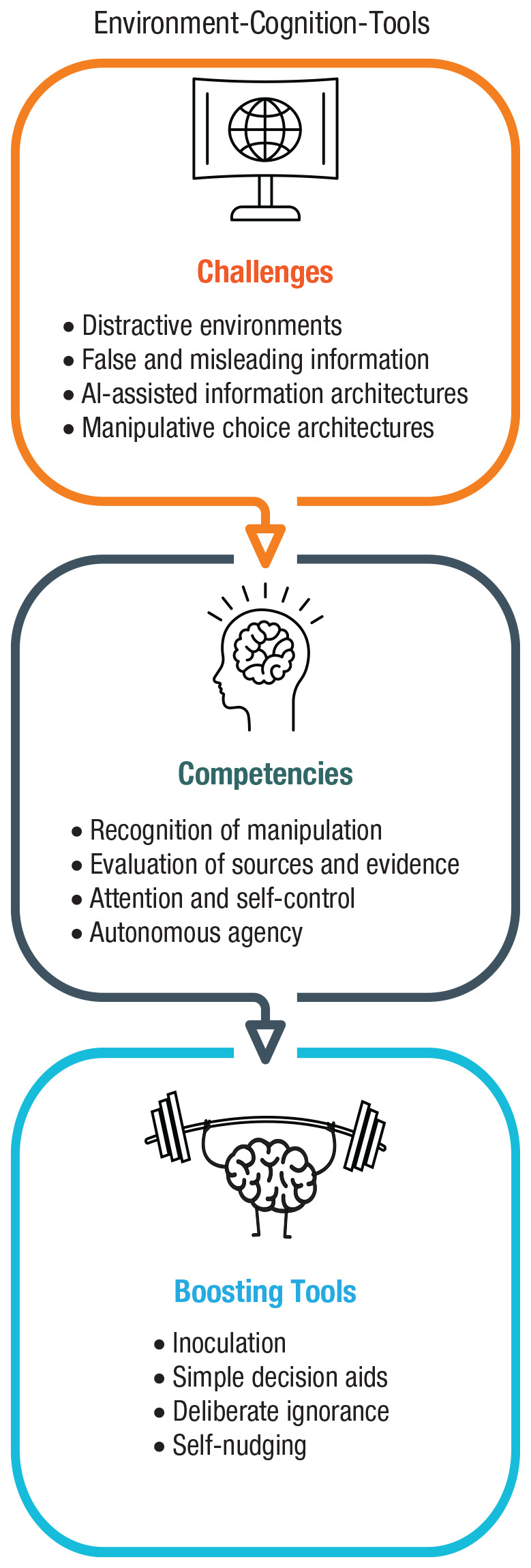
Map of challenges and boosts in the digital world. Icons are used under license from Adobe Stock.

The focus of this article has been on challenges that threaten people’s agency, their choice autonomy, and the epistemic quality of their information environment. Many other challenges exist and new ones are quickly emerging, such as the massive amounts of highly plausible but fabricated video and audio material known as *deepfakes* that are further deflating confidence in a shared reality. In an increasingly “onlife” world (Floridi, 2014), behavioral sciences, social sciences, law, computer science, and—we believe—psychological science in particular face important tasks. One is to measure and understand the psychological effects of these revolutionary transformations. Another is to develop and design policy interventions that help people cope with the consequences of those transformations. Focusing on the four challenges of online environments, we outlined various classes of interventions that are informed by the behavioral sciences, then focused in on interventions aimed at empowering people; returning a sense of agency to people (e.g., the citizen as a choice architect); and fostering autonomy, self-control, and resistance to being manipulated in the digital world—in other words, interventions meant to cultivate a sense of self-efficacy and ontological security (see Fig. 13).

These four types of tools can also be summarized as four simple rules for mindful Internet behavior that could become as routine as washing one’s hands or checking for cars before crossing the street:

1. Control and organize your digital environment. Adapt it to your goals.
2. Learn to ignore and filter out nonessential and misleading information.
3. Make a habit of using simple rules for data privacy and information literacy.
4. Immunize yourself against the most common and dangerous types of manipulation and dis-information.

For policy makers, these rules can mean:

1. Ensure that users have adequate control over their digital environments and personal data. Make it easy for them to take the reins.
2. Make it easy for people to separate useful information from noise and disinformation—for instance, by mandating clear, intuitive indicators of epistemic quality.
3. With the help of researchers, design simple rules for data privacy and information literacy and provide them to users.
4. Monitor common types of online disinformation and manipulation and provide appropriate and timely inoculations.

We have no illusions. There is no single solution for these and many other challenges. It is very likely that these interventions will be shown to have some benefits, but only for some users. Nevertheless, it is important to start, and soon: Several surveys show that people are concerned about data privacy, the spread of false information, political manipulation, and online harassment (e.g., Directorate-General for Communication, 2018; Kozyreva et al., 2020; Mitchell et al., 2019; Newman et al., 2020). Any solution will require the orchestrated efforts of regulators, policy makers, educators, and users—for instance, boosting people’s ability to control the default parameters of their choice architectures should be accompanied by a regulatory framework that takes the heterogeneity of users into account. Specifically, the law could be used to prevent companies from taking advantage of the fact that some citizens fail to take control of their default privacy settings (e.g., the EU’s GDPR, embracing insights from the behavioral sciences, mandates that data controllers can no longer use opt-out as a default for obtaining consent to data processing).

The rules and design of Internet landscapes are predominantly dictated by major corporations and signal a lack of a coherent regulatory framework for transparent and robust user protection. Contrary to promises of the early digital era (e.g., access to information for all, empowered minorities, unsuppressed democratic deliberation), citizens find themselves in a state of constant information overload, surveillance, manipulation, and digital divide. We believe that psychological science must contribute to the long-term goal of designing and fostering the “Internet for citizens,” an online world respectful of fundamental human rights and values that will require users to learn new competencies and make active decisions. One may think this is an unrealistic ideal. Yet one need look no further than the digital world itself for evidence of the spectacular human ability to learn: Fifty years ago, Neil Armstrong became the first person to step onto the moon. And yet, at that time, it was hard to imagine with what ease a 9-year-old of today could navigate the digital world.

## Transparency

*Editor:* Nora Newcombe

## Notes

1. The present regulatory framework is fragmented and disparate and focuses on the types of actors online instead of providing a more coherent form of protection that covers the entirety of the online experience (Leiser, 2019). Moreover, the EU and the United States are likely to pursue different regulatory approaches to specific problems because the First Amendment to the U.S. Constitution will take precedence over many other goals (e.g., privacy).

2. Ubiquitous computing describes technology that, by virtue of its pervasiveness in everyday life, has become invisible to people (Weiser, 1991).

3. McFarland and Ployhart (2015) outlined eight discrete ambient stimuli that distinguish social-media contexts from nondigital contexts: physicality, accessibility, latency, interdependence, synchronicity, permanence, verifiability, and anonymity.

4. Meshi et al. (2015) proposed six features that affect the ways users interact: user identity, information format, text length, network connections, spatial distance, and temporal scale (p. 7).

5. See Statista (2020a) and Columbus (2016). Although digital technologies are increasingly accessible, almost half of the world’s population, mainly in low-income regions, does not yet have access to the Internet. This striking inequality also extends to content production, which is mostly generated in developed countries across Europe and North America: “The global North is characterized by the greatest levels of participation and is creating the bulk of digital content, while the global South contributes very little. Africa, in particular, is almost entirely omitted from these processes of digital generativity” (Graham et al., 2015, p. 97).

6. The cognitive limit on the size of natural face-to-face social networks is thought to be determined by a combination of constraints on available social time and cognitive bounds. According to this approach (the social-brain hypothesis; Dunbar, 1998), cognitive constraints are related to the size of the neocortex and associated information-processing capacity that, in primates, correlates closely with the typical size of social groups. In humans, this suggests a social circle of about 150 people, with hierarchical levels reflecting both emotional closeness and interaction frequency. These layers have values that approximate five (closest friends, or “support clique”), 15 (“sympathy group”), 50, and 150, and extend beyond this in at least two further layers to 500 and 1,500 (Dunbar, 2016).

7. These numbers are based on estimates from https://www.internetlivestats.com.

8. It was a tweet by Japanese billionaire Yusaku Maezawa, who promised on January 5, 2019, to give away 100 million yen ($924,000) to be shared among 100 random people (Brandwatch, 2020).

9. “This means not just demographic or profiling data, but also behavioural metadata, such as those automatically derived from smartphones, like time stamps and GPS-inferred locations” (Kennedy et al., 2015, para. 2).

10. Further examples can be found at https://www.darkpatterns.org/types-of-dark-pattern.

11. A counterargument to the objective of algorithmic transparency is the potential for misuse and manipulation if their inner workings were made public—for instance, “Google bombing,” when web pages are artificially overlinked in order to push them higher up in search results. Trolls and bots can also game algorithmic filters by flooding social media with posts, thereby amplifying the posts in news feeds. However, these tactics do not depend on transparency—indeed, they are already in use. Furthermore, compared with current, hidden designs, transparency and accountability can make algorithms less vulnerable to exploitation.

12. See Statista (2020b); Alexa (2020).

13. We are not aware of any analyses pertaining to Europe.

14. Lewandowsky, Jetter, and Ecker (2020) showed that the agenda-setting power of misleading news also extends to the American president’s tweets. In response to media coverage he considered threatening (e.g., the Mueller investigation), President Trump strategically deployed distraction (e.g., tweeting about China, jobs, or immigration) to set the agenda for the mainstream media (e.g., *The New York Times* and ABC News), thus prompting less coverage of the issues he wanted removed from public discourse.

15. Although perceived exposure to false information can differ from the actual encounters with unverified and misleading claims, perceived prevalence of falsehoods online can have tangible consequences of increasing mistrust in the media and political institutions (e.g., CIGI-Ipsos, 2019).

16. Reference to “disorder” in this context does not mean that the blame is shifted to the user but rather that information disorders are not there by design; they emerge as malfunctions in a healthy flow of information.

17. Note that the content considered “true” in this study was fact-checked content determined to be true; this is not representative of the broader category of “truth,” which includes legitimate news stories that have not been fact-checked. In a similar vein, the study examined the characteristics of the fake news that indeed spreads, rather than the false stories that do not become as successful in spreading on social networks but stay unnoticed. We thank Gordon Pennycook and one anonymous reviewer for pointing out these two important limitations of the cited study.

18. The same study found that attentional capture is associated with retweet behavior in online moral and political discourse: Tweets with a greater attention-capture value (as assessed by specific words in the tweet) were retweeted more often (Brady et al., 2019).

19. Unfortunately, people are generally not particularly skilled at evaluating the trustworthiness of information they encounter online. Research in education suggests that young people struggle with many aspects of finding reliable information online—from selecting search results to judging whether a site is trustworthy. Students tend to ignore the source of information, have difficulties distinguishing between traditional news and sponsored content, and evaluate sites on the basis of superficial features such as graphic design and authoritative logos (Barzilai & Zohar, 2012; McGrew et al., 2018; Wiley et al., 2009; Wineburg & McGrew, 2019; Wineburg et al., 2016). The same dismissal of important information about the source of online news has been observed in a large sample of adult participants (Dias et al., 2020). In a similar vein, only 9% of participants in Amazeen and Wojdynski’s (2018) study were able to distinguish advertising from editorial content. This ability seems to decline with age. However, when asked about trustworthiness of different news domains, people can successfully distinguish mainstream media outlets from hyperpartisan and fake-news websites (Pennycook & Rand, 2019a).

20. The evidence, however, is mixed: The link between multitasking and cognitive control (distractibility) turns out to be weaker than previous studies have suggested (for a replication study and a meta-analysis, see Wiradhany & Nieuwenstein, 2017). The general discrepancy in findings is also highlighted in the literature review on the minds and brains of media multitaskers by Uncapher and Wagner (2018). At the same time, the authors of the review concluded that in light of the current evidence (both convergent and divergent), heavier media multitaskers exhibit poorer performance in a number of cognitive domains (e.g., working memory; Uncapher & Wagner, 2018).

21. The data sets were from Instagram and three topic-based social networks.

22. According to Smith (2018a): Half (50%) of Facebook adopters who think users have a lot of control over their news feeds have themselves attempted to influence the content they see there. But that share falls to 40% among those who think users have only a little control over the content of their feeds, and to 24% among those who think they have no control. (para. 5)

23. Rose-Stockwell (2018) argued that social media could provide users with curation tools for their own algorithmic filtering, such as prioritizing posts from family members, diversifying the spectrum of political news shown, ordering news chronologically, or filtering out posts filled with moral outrage. MIT Social Media Lab has developed Gobo, a social-media aggregator with filters that users can control: https://www.media.mit.edu/projects/gobo/overview/.

24. Some have suggested that the deliberate use of heuristics (i.e., cognitive strategies that ignore part of the available information) is also an instance of deliberate ignorance. Hertwig and Engel (2020) have argued on conceptual grounds against this interpretation. Furthermore, there is an ongoing debate about whether voters’ knowledge of political facts (e.g., candidates’ positions on various issues) is a good proxy for the quality of their decisions, given that heuristics that foster good decisions using only a limited amount of information may compensate for a lack of political sophistication (e.g., Lupia & McCubbins, 1998; but see Lau & Redlawsk, 2001). Outside the context of voter decision-making, there is ample evidence for the success of ecologically rational heuristics that rely on limited information (see Gigerenzer et al., 2011; Hertwig et al., 2019). The success of heuristics depends on reasonably accurate cue information. One of the cognitive tools that allows people to curate their knowledge base is informed deliberate ignorance.

25. For instance, online narratives of parents who report adverse effects of vaccines appear to be an important source of doubt about vaccination safety (the “narrative bias”; Betsch et al., 2015; Haase & Betsch, 2012).

26. There is an accessible computerized toolbox for creating, visualizing, and evaluating FFTs called FFTrees (Phillips et al., 2017).

27. Like vaccines, which use a weakened version of a virus, a weakened version of disinformation does not entail the same level of risk as encountering such disinformation in an uncontrolled setting might.
